# A fat- and sucrose-enriched diet causes metabolic alterations in mdx mice

**DOI:** 10.1152/ajpregu.00246.2022

**Published:** 2023-10-09

**Authors:** Swathy Krishna, Kenneth G. Echevarria, Carter H. Reed, Hyeyoon Eo, Michelle Wintzinger, Mattia Quattrocelli, Rudy J. Valentine, Joshua T. Selsby

**Affiliations:** ^1^Department of Animal Science, Iowa State University, Ames, Iowa, United States; ^2^Department of Kinesiology, Iowa State University, Ames, Iowa, United States; ^3^Division of Molecular Cardiovascular Biology, Heart Institute, Cincinnati Children’s Hospital Medical Center, Cincinnati, Ohio, United States; ^4^Department of Pediatrics, University of Cincinnati College of Medicine, Cincinnati, Ohio, United States

**Keywords:** C57, DMD, high-fat diet, metabolism, obesity

## Abstract

Duchenne muscular dystrophy (DMD), a progressive muscle disease caused by the absence of functional dystrophin protein, is associated with multiple cellular, physiological, and metabolic dysfunctions. As an added complication to the primary insult, obesity/insulin resistance (O/IR) is frequently reported in patients with DMD; however, how IR impacts disease severity is unknown. We hypothesized a high-fat, high-sucrose diet (HFHSD) would induce O/IR, exacerbate disease severity, and cause metabolic alterations in dystrophic mice. To test this hypothesis, we treated 7-wk-old mdx (disease model) and C57 mice with a control diet (CD) or an HFHSD for 15 wk. The HFHSD induced insulin resistance, glucose intolerance, and hyperglycemia in C57 and mdx mice. Of note, mdx mice on CD were also insulin resistant. In addition, visceral adipose tissue weights were increased with HFHSD in C57 and mdx mice though differed by genotype. Serum creatine kinase activity and histopathological analyses using Masson’s trichrome staining in the diaphragm indicated muscle damage was driven by dystrophin deficiency but was not augmented by diet. In addition, markers of inflammatory signaling, mitochondrial abundance, and autophagy were impacted by disease but not diet. Despite this, in addition to disease signatures in CD-fed mice, metabolomic and lipidomic analyses demonstrated a HFHSD caused some common changes in C57 and mdx mice and some unique signatures of O/IR within the context of dystrophin deficiency. In total, these data revealed that in mdx mice, 15 wk of HFHSD did not overtly exacerbate muscle injury but further impaired the metabolic status of dystrophic muscle.

## INTRODUCTION

Duchenne muscular dystrophy (DMD) is a progressive muscle disease that affects 1 in 3,500 to 6,000 boys born worldwide (1, 2). DMD is caused by the absence of functional dystrophin protein, a major structural component of the dystrophin-glycoprotein complex (DGC). Dystrophin provides a link between the cytoskeleton and the extracellular matrix through the DGC. Dystrophin deficiency leaves muscle susceptible to contraction-induced injury and subsequent fibrosis, fatty infiltration, and inflammation and causes multiple secondary cellular dysfunctions including, but not limited to, oxidative stress, increased proteolysis, mitochondrial dysfunction, impaired autophagy, and calcium dysregulation (3–11). The DGC also plays a crucial role in signal transduction for important cellular pathways, including insulin signaling (12, 13).

Obesity and related metabolic defects, including insulin resistance (IR), have been previously reported in boys/men with DMD at multiple stages of disease progression (14–17). Although the precise mechanisms leading to obesity and/or IR (O/IR) have not been elucidated, it is likely that reduced mobility, decreased energy expenditure, and overfeeding contribute to the development of obesity and associated metabolic alterations commonly found in DMD (18, 19). Alterations in gastrointestinal function associated with DMD may also increase the risk of metabolic defects (20). In addition, glucocorticoids are frequently used to treat boys with DMD, and while the evidence of the overall efficacy of glucocorticoids is overwhelming, they may also contribute to O/IR (21–24). Despite the high frequency of O/IR as a comorbidity with DMD, the extent to which O/IR modifies metabolism and disease severity is poorly understood.

In contrast to boys/men with DMD, the mdx mouse model, the most extensively researched mouse model of DMD, is exceedingly lean and has a notably mild disease phenotype (25–27). Remarkably, an early attempt to drive obesity using a 16% fat diet decreased disease severity (28). In contrast, genetic ablation of ApoE in combination with a Western diet (0.2% cholesterol, 21% fat, and 34% sucrose) induced dyslipidemia and increased myofiber damage and fibrofatty replacement in gastrocnemius (GAS) and triceps brachii from a rodent model of DMD (29). These studies support a rationale that diet can impact disease progression, but the extent to which diet-induced O/IR may modify disease phenotype or metabolotype remains largely unknown.

The purpose of this investigation was to determine the extent to which a high-fat, high-sucrose diet (HFHSD) would impact disease pathology in mdx mice. An additive or even synergistic effect of O/IR and dystrophin deficiency may stem from overlapping and independent cellular dysfunctions caused by each condition. Since the pathological modifications due to diet-induced O/IR may increase disease progression and severity, this may provide insight into underlying mechanisms contributing to disease progression in humans and may also help recapitulate human disease severity more faithfully in the mdx mouse model. We hypothesized that an HFHSD would induce O/IR, exacerbate disease severity, and cause metabolic alterations in dystrophic mice.

## METHODS

### Animal Treatments

All animal procedures were approved by the Institutional Animal Care and Use Committee at Iowa State University. Male C57BL/6J (C57, wild-type control) and C57BL/10ScSn-*Dmd^mdx^*/J (mdx, DMD model) mice were obtained from Jackson Laboratories. Mice were received at 6 wk of age and were allowed to acclimate for 1 wk before study initiation. Mice were housed in conventional cages initially with 3–4 mice per cage with selected housing changes due to aggressive behaviors made throughout the study period as necessary. Starting at 7 wk of age, randomly selected C57 and mdx mice (*n* = 6/group) were maintained on a control diet (D12450K Research Diets, Inc.) composed of 20% protein, 10% fat, and 70% carbohydrate (no sucrose) at an energy density of 3.82 kcal/g. Alternatively, to cause diet-induced obesity, C57 and mdx mice (*n* = 6/group) were fed an HFHSD (D12451 Research Diets, Inc.) composed of 20% protein, 45% fat, and 35% carbohydrate (sucrose constituting 20% of the total diet by weight), matched for all other nutrients, providing an energy density of 4.7 kcal/g. All mice were fed ad libitum on assigned diets for 15 wk and maintained on a reversed light-dark cycle, followed by tissue collection at 22 wk of age. Total food intake for each cage was recorded twice weekly and the average intake for each animal was calculated. Individual body weights were recorded twice weekly throughout the study. At the end of the study period, mice were brought to a surgical level of anesthesia using pentobarbital (60 mg/kg) for tissue [limb muscles, diaphragm, epididymal adipose (EPI), and perirenal adipose depots] and blood collection, and ultimately euthanized via removal of the diaphragm.

### Glucose Tolerance Test and Insulin Tolerance Test

To assess insulin sensitivity, mice were subjected to a glucose tolerance test (GTT) and insulin tolerance test (ITT). To prevent the GTT from interfering with the ITT, these tests were separated by 2 wk. Similarly, to prevent the possibility of impacting end point measures, the ITT was performed 2 wk before tissue collection. Hence, the GTT was performed following 10 wk of treatment. Mice were fasted overnight and then given an intraperitoneal glucose injection at 2 g/kg body weight. Blood glucose concentration was measured with a glucometer (OneTouch Ultra2 blood glucose monitoring system, product number 5388500027) using blood collected from the tail vein before (0 min) and 30, 60, 90, and 120 min after glucose delivery. The area under the curve (AUC) was calculated for blood glucose using GraphPad prism software.

Insulin tolerance tests were performed following 12 wk of treatment by intraperitoneal injection of insulin at a dosage of 0.25 IU/kg body wt after 2 h of fasting. Blood glucose concentration was measured as aforementioned using blood from the tail vein before (0 min) and 15, 30, 60, and 90 min following insulin delivery. The rate of glucose disappearance (Kitt) was calculated using the equation Kitt = $\frac{\text{slope of glucose clearance}\times 100}{\text{basal glucose level}}$, where the slope of glucose clearance is obtained by fitting a linear regression equation for the glucose concentration from 0 to 30 min (30–32), after which blood glucose started to rise, ostensibly due to glucagon (30).

C57 mice used herein were part of a larger cohort of mice serving several experimental objectives (*n* = 24 lean; *n* = 24 obese). Twelve mice from each group were randomly selected for a GTT and 12 mice were randomly selected for an ITT before assignment to this investigation; hence ITT and GTT have *n* = 12/group for C57, whereas the remaining measures will use *n* = 6/group. ITT and GTT were performed in random order, and treatment groups were completed in a preset order such that all groups were represented on each day of data collection.

### Lipidomics

Blood collected on euthanasia was allowed to clot at room temperature for 30 min and then centrifuged at 2,000 *g* for 10 min at 4°C. The resulting supernatant, which is the serum, was transferred into fresh tubes and stored at −80°C until use. Stored serum extracts (10 µL) were used for sample extraction. Internal standard (30 µL) was added to 10 µL serum/sample aliquot in a disposable culture tube. Butanol/methanol (300 µL; 3:1) was added to the sample aliquot tube and vortexed for 10 min, followed by 150 mL of heptane/ethyl acetate (3:1) to the sample aliquot tube and vortexed for 5 min. The contents were then transferred to a large glass culture tube and 150 mL of heptane/ethyl acetate (3:1) was added and vortexed for 5 min. The contents were then transferred to a large glass culture tube. Then 300 mL of 1% acetic acid was added to the tube contents, vortexed for 5 min, allowed to settle for 5 min, and 200 mL of the upper organic phase was then transferred to a small glass culture tube. Next, 320 mL of heptane/ethyl acetate (3:1) was added to the water phase, vortexed for 5 min, and allowed to settle for 5 min (if the phases did not separate, centrifuge for 6 min at 2,000 *g*). The upper organic phase (280 µL) was combined with the first extract in the small glass tube. This step was repeated once more to obtain an optimum amount of extract. The combined extracts were transferred to the Mass Spectrometry Core at the Cincinnati Children’s Hospital Medical Center for drying and analysis. An untargeted lipidomics analysis was performed under blinded conditions on a Q Exactive plus hybrid quadrupole-Orbitrap mass spectrometer interfaced with Vanquish ultra-high performance liquid chromatography (UHPLC) system (Thermo Fisher Scientific) following data acquisition and lipid annotation, according to previously described methods (33). The lipid species were classified into different categories, main classes, and subclasses based on the classification system established in LIPID MAPS (34).

### Histology

The diaphragm was divided into a right and left hemidiaphragm upon tissue collection. One randomly selected hemidiaphragm from each animal was fixed in paraformaldehyde, mounted in paraffin, and sectioned to obtain 10-µm thick cross-sections for histological analysis.

To perform trichrome staining, slides were deparaffinized by serial washes in CitriSolv (3 washes for 5 min each; Decon Labs, Inc., 1601) followed by rehydration in 100% ethanol (2 washes, 1 min each) and 70% ethanol (1 wash, 1 min) and a 1 min wash in distilled water. The slides were incubated in Bouin’s solution (Sigma Aldrich, HT15) overnight at room temperature and washed in running tap water until colorless. Nuclear staining was obtained by immersing the slides in Weigert’s hematoxylin (Sigma-Aldrich, HT15) for 8 min, followed by a 5-min wash in tap water. After the slides were rinsed in distilled water, the cytoplasm was stained by incubating slides in Biebrich Scarlet acid Fuchsin (Sigma-Aldrich, HT15) for 8 min. The excess stain was removed by rinsing in distilled water. To stain collagen, slides were then immersed in phosphotungstic-phosphomolybdic acid solution (Sigma-Aldrich, HT15) for 8 min followed by aniline blue (Sigma-Aldrich, HT15) for 5 min, and then 1% acetic acid solution for 3 min. Slides were washed using 10 serial dips in 95% ethanol (2 washes) and 100% ethanol (2 washes) followed by 3 CitriSolv washes. Coverslips were mounted with permount, and slides were sealed with nail polish.

Trichrome-stained sections were imaged in identified samples at ×20 with an inverted DMI3000 B microscope and QICAM MicroPublisher 5.0 (MP5.0-RTV-CLR-10, QIMAGING) camera using QCapture software. Three random, nonoverlapping, ×20 images were captured from each section and were objectively quantified using OpenLab 3.5.1 software. The total cross-sectional area of each section was determined, and the percent fibrosis was calculated by quantifying the blue-stained areas in the images and expressing them relative to the total cross-sectional area. Likewise, the contractile area was quantified by measuring the red-stained area (excluding fibrosis and nuclei) and expressing it relative to the total cross-sectional area.

### Western Blot

Western blot was performed as previously described (5). Briefly, whole muscle protein was extracted from the contralateral hemidiaphragm using whole muscle buffer (10 mM sodium phosphate buffer, pH 7.0, 2% SDS, 1% Halt protease, and phosphatase inhibitor single-use cocktail) added at a 1:10 ratio of tissue weight to buffer volume and centrifuged at 10,640 *g* for 10 min. Protein from a nuclear fraction was also isolated, as previously described (35) using NE-PER Nuclear and Cytoplasmic Extraction Reagents from Thermo Fisher according to manufacturer’s instructions. Briefly, frozen tissue was powdered and homogenized in ice-cold CER II (cytoplasmic extraction reagent) and vortexed for 5 s, incubated on ice for 1 min, vortexed again, and then centrifuged at 16,000 *g* for 5 min. The supernatant was discarded, and the insoluble pellet was suspended in ice-cold NER and vortexed for 15 s every 10 min for 40 min. The samples were then centrifuged at 16,000 *g* for 10 min. The supernatant, which is the nuclear extract, was transferred to a clean, prechilled tube. The nuclear fraction was stored at −80°C until use. A working concentration of 3 µg/µL of protein was used for Western blots.

Protein was separated at 180 V for 45 min using 4–20% Criterion Protein Gels (26 well) and transferred to nitrocellulose membranes at 100 V for 1 h at 4°C. Lane allocation for Western blots was predetermined to have a replicating pattern cycling through the groups; however, the sample within a group was randomly selected for a group-assigned lane. After transfer, the membranes were stained with Ponceau S stain, imaged using Azure C600, and objectively quantified using whole lane quantification with AzureSpot software to confirm equal loading. Ponceau S stain was removed by two washes in Tris-buffered saline with 0.1% Tween 20 (TBST, 2 min each), and membranes were blocked in 5% nonfat dehydrated milk in TBST for 1 h, followed by two TBST (2 min each) washes. The membranes are then incubated in primary and secondary antibodies as indicated in Tables 1 and 2 with three, 10-min washes in TBST between antibody treatments. After three 10-min washes in TBST, membranes were exposed to ECL for 5 min and then imaged using Azure C600. Bands were objectively quantified using AzureSpot software with automated band detection to limit bias (5).

**Table 1.** Antibodies used in Western blotting of whole muscle extract from the diaphragm

Antibody	Product Details	Company	Host	Primary	Secondary
AMPK	5832S	Cell Signaling	Rabbit	1:1,000 TBST	1:2,000 TBST
AP1	A5968	Sigma	Rabbit	1:1,000 TBST	1:2,000 TBST
ATG12-5	4180S	Cell Signaling	Rabbit	1:1,000 TBST	1:2,000 TBST
CTSB	31718	Cell Signaling	Rabbit	1:1,000 TBST	1:2,000 TBST
LAMP1	9091	Cell Signaling	Rabbit	1:1,000 TBST	1:2,000 TBST
LAMP2	49067	Cell Signaling	Rabbit	1:1,000 TBST	1:2,000 TBST
LC3A/B	12741	Cell Signaling	Rabbit	1:1,000 TBST	1:2,000 2.5% milk
mTOR	2972	Cell Signaling	Rabbit	1:1,000 TBST	1:2,000 TBST
NFκB	8242	Cell Signaling	Rabbit	1:1,000 TBST	1:2,000 TBST
OXPHOS	AB110413	Abcam	Mouse	1:1,000 TBST	1:2,000 TBST
p62	AB91526	Abcam	Rabbit	1:500 2.5% milk	1:1,000 TBST
pAMPK T172	2535	Cell Signaling	Rabbit	1:1,000 TBST	1:2,000 TBST
Perilipin 2	NB110-40877	Novus Biologicals	Rabbit	1:1,000 TBST	1:2,000 5% milk
PGC1α	20658-1-AP	Protein-tech	Rabbit	1:1,000 TBST	1:2,000 TBST
pJNK T183/185	9251	Cell Signaling	Rabbit	1:750 TBST	1:2,000 TBST
PKR	SC-6282	Santa Cruz	Rabbit	1:1,000 TBST	1:2,000 TBST
pmTOR S2448	2971	Cell Signaling	Rabbit	1:1,000 TBST	1:2,000 TBST
pPKR T446	AB32036	Abcam	Rabbit	1:1,000 TBST	1:2,000 TBST
pULK1 S555	5869	Cell Signaling	Rabbit	1:1,000 TBST	1:2,000 TBST
SESN2	8487	Cell Signaling	Rabbit	1:1,000 TBST	1:2,000 TBST
TFAM	8076	Cell Signaling	Rabbit	1:500 TBST	1:1,000 TBST
TLR4	SC-293072	Santa Cruz	Mouse	1:1,000 TBST	1:2,000 TBST
TNFα	MP6-XT22	Invitrogen	Mouse	1:1,000 TBST	1:2,000 TBST
UCP2	SC-390189	Santa Cruz	Mouse	1:1,000 TBST	1:2,000 TBST
ULK1	8054	Cell Signaling	Rabbit	1:500 TBST	1:1,000 5% milk
IKKα	2682	Cell Signaling	Rabbit	1:1,000 TBST	1:2,000 TBST
IKBα	4812	Cell Signaling	Rabbit	1:1,000 TBST	1:2,000 TBST

**Table 2.** Antibodies used in Western blotting of nuclear fraction from the diaphragm

Antibody	Product Details	Company	Host	Primary	Secondary
PGC1α	20658-1-AP	Protein-tech	Rabbit	1:1,000 TBST	1:1,000 5% milk
TFAM	8076	Cell Signaling	Rabbit	1:500 TBST	1:1,000 5% milk
NFκB	8242	Cell Signaling	Rabbit	1:1,000 TBST	1:2,000 TBST
AP1	A5968-2ML	Sigma	Rabbit	1:1,000 TBST	1:2,000 5% milk
KEAP1	8047	Cell Signaling	Rabbit	1:1,000 TBST	1:1,000 5% milk
NRF2	12721	Cell Signaling	Rabbit	1:1,000 5% milk	1:2,000 TBST

### Metabolomics

The metabolite extraction from quadriceps (QUAD) muscles was performed using a previously established method (36). Ribitol (10 μL from 1.2 mg/mL stock in water) was added to 60–90 mg of the powdered, frozen muscle tissue as an internal standard for polar compounds. In addition, 10 μL of nonadecanoic acid (NAA; from 1.3 mg/mL stock in hexane) was added to samples as an internal standard for nonpolar compounds. Then, 0.5 mL of ice-cold methanol was added to the samples, vortexed for 3 min, and placed into an ice-cold sonication water bath for 10 min at full output power. Two, 2.4-mm metal beads were added to the samples; the tissue was then homogenized using a Bead Mill 24 Homogenizer (Thermo Fisher Scientific, Inc., Waltham, MA). Next, 0.5 mL of chloroform was added to the samples and vortexed for 3 min followed by the addition of 0.35 mL of water and another 3 min vortex before the samples again were placed into an ice-cold sonication water bath for 10 min at full output power. The samples were vortexed for 3 min and then centrifuged for 7 min at maximum speed (16,000 *g*). Three-hundred microliters of the upper layer (polar fraction) and 200 μL of the lower layer (nonpolar fraction) were added to GC-MS vials. Samples were dried in a speed-vac concentrator for 10 h and then derivatized (37). Fifty microliters of methoxyamine hydrochloride (20 mg/mL in pyridine) were added to the dried polar extract, followed by a 1.5 h incubation at 30°C. Trimethylsilylation (TMS) was performed by the addition of 70 μL of bis-trimethyl silyl trifluoroacetamide with 1% trimethylchlorosilane (BSTFA + 1% TMCS) for 30 min at 60°C.

Derivatized samples were analyzed by GC-MS at the ISU W.M. Keck Metabolomics Research Laboratory under blinded conditions. GC-MS analyses were performed with an Agilent 6890 gas chromatograph coupled to a model 5973 Mass Selective Detector (Agilent Technologies, Santa Clara, CA). The column used was HP-5MS 5% phenyl methyl silox with 30 m × 250 µM × 0.25 µm film thickness (Agilent Technologies). One microliter of the sample was injected with the inlet operating in splitless mode and held at a constant temperature of 280°C. The oven temperature was programmed as follows: an initial temperature of 70°C was increased to 320°C at 5 °C/min and held for 8 min. Helium was used as a carrier gas at a flow rate of 1 mL/min. The MS transfer line was held at 280°C. Mass spectrometry detection was performed using electron ionization at 70 eV, and source temperature and quadrupole temperature were set at 230°C and 150°C, respectively. The mass data were collected in the range from *m/z* 40 to *m/z* 800. Identification and quantification were conducted using AMDIS [Automated Mass spectral Deconvolution and Identification System, National Institute of Standards and Technology (Gaithersburg, MD)] with a manually curated retention indexed GC-MS library with additional identification performed using the NIST17 and Wiley 11 GC-MS spectral library (Agilent Technologies, Santa Clara, CA). Final quantification was calculated by integrating the corresponding peak areas relative to the area of the internal standards. Raw data were normalized to the mass of tissue used.

### Statistics

Sample size was estimated with power calculations using anticipated effect size and variability for epididymal fat mass, GTT AUC, and Homeostatic Model Assessment for Insulin Resistance (HOMA-IR) values based on our previous work (38). Normality was confirmed in all measures using the Shapiro–Wilk test before the statistical testing. For end point measures, a two-way ANOVA with multiple comparisons with Tukey adjustments or an unpaired *t* test (when required) was performed using GraphPad PRISM (v.8.2.0). The GTT and ITT were analyzed using repeated-measures ANOVA in R. Significance was established at *P* < 0.05. For the lipidomics data analysis, including differential expression, categorization, and visualization (39), the following R packages were used: POMA (40), tidyverse (41), car (42), emmeans (43), and ggplot (44). Statistical evaluation of the nontargeted GC-MS data from metabolomics was conducted with the R-based statistical package, POMA (40) and MetaboAnalyst (45). MetaboAnalyst’s multivariate analysis tools were used for pathway and localization enrichment analyses. MetaboAnalyst 5.0 (45) and POMA (R-package) (40) were used for analyses and data visualizations. Venn diagrams were created using jvenn (46). Data points more than two standard deviations from the mean were identified as outliers and removed from the data set regardless of group or direction.

## RESULTS

### Body Weight, Feed Intake, and Caloric Consumption

**Figure 1.**
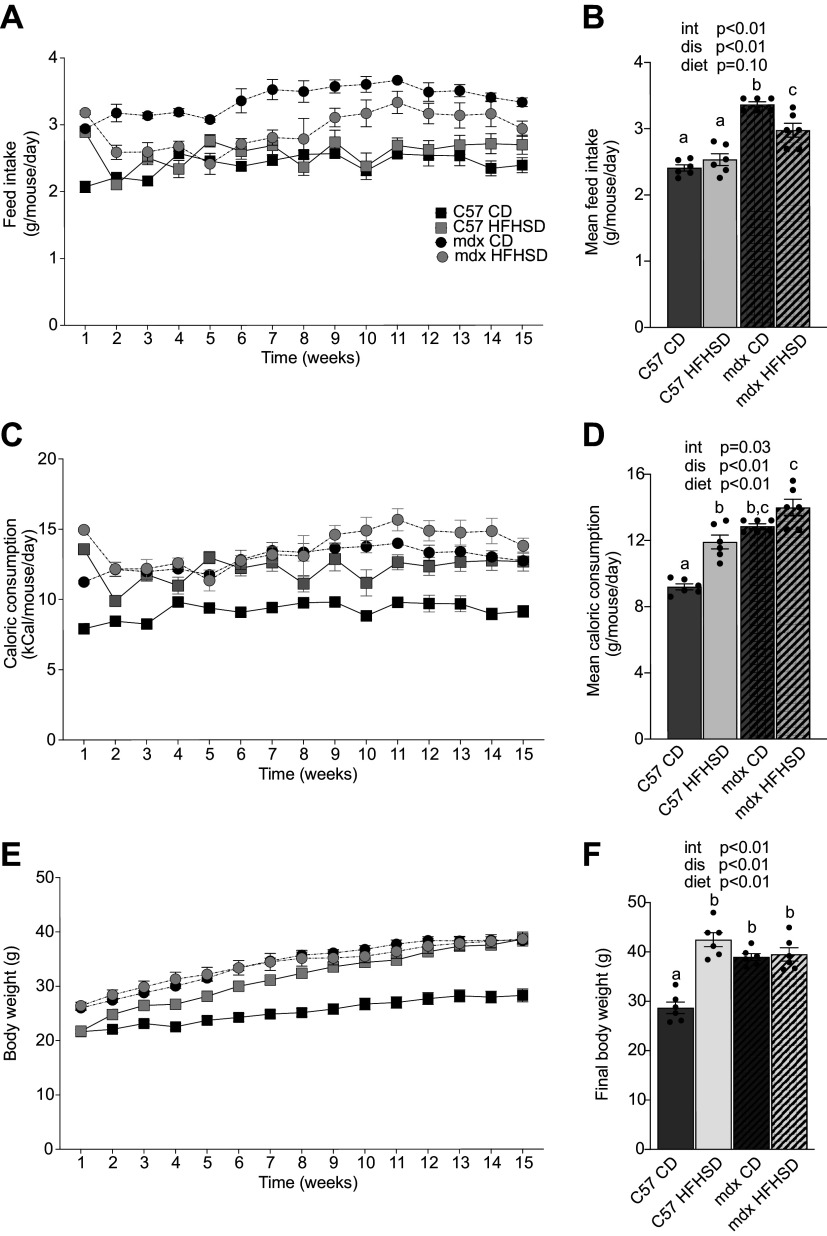
A differential effect of a high-fat, high-sucrose diet (HFHSD) in C57 and mdx mice. Body weight, caloric consumption, and feed intake were assessed weekly for 15 wk in both C57 and mdx mice starting at 7 wk of age. *A* and *B*: feed intake was measured weekly and is expressed in grams per mouse per day. *C* and *D*: weekly caloric consumption measured in kilocalories per mouse per day. *E*: body weight expressed in grams plotted over time. *F*: final body weight was considered individually. Main effects (dis, disease; diet; and int, interaction) from two-way ANOVA are indicated above each graph. Means ± SE is represented. Significance established at *P* < 0.05. Different lowercase letters indicate differences between groups (*n* = 6 mice/group for all groups).

Average daily feed intake was increased as a main effect of disease (*P* < 0.0001), and mdx CD was greater than mdx HFHSD (*P* = 0.0002, Fig. 1). Average daily caloric consumption was increased as a main effect of disease (*P* < 0.0001) such that mdx consumed more calories than C57, and as a main effect of diet (*P* < 0.0001) such that the HFHSD-fed mice consumed more calories than CD-fed mice. There was also a larger increase in caloric consumption in the C57 HFHSD compared with C57 CD than the corresponding mdx groups and a significant interaction (*P* = 0.0339). Of note, the mdx HFHSD ate more calories/day than the C57 HFHSD, and the mdx CD and mdx HFHSD consumed similar calories/day. This caloric pattern was reflected in final body weight such that C57 and mdx HFHSD-fed groups and the mdx CD group were greater than C57 CD (*P* < 0.05; Fig. 1). There were main effects of disease such that the body weight of mdx mice was greater than C57 and a main effect of diet such that mice fed a HFHSD were greater than mice fed CD, though this was driven by the large change in body mass in C57 HFHSD compared with C57 CD.

### Insulin Resistance

**Figure 2.**
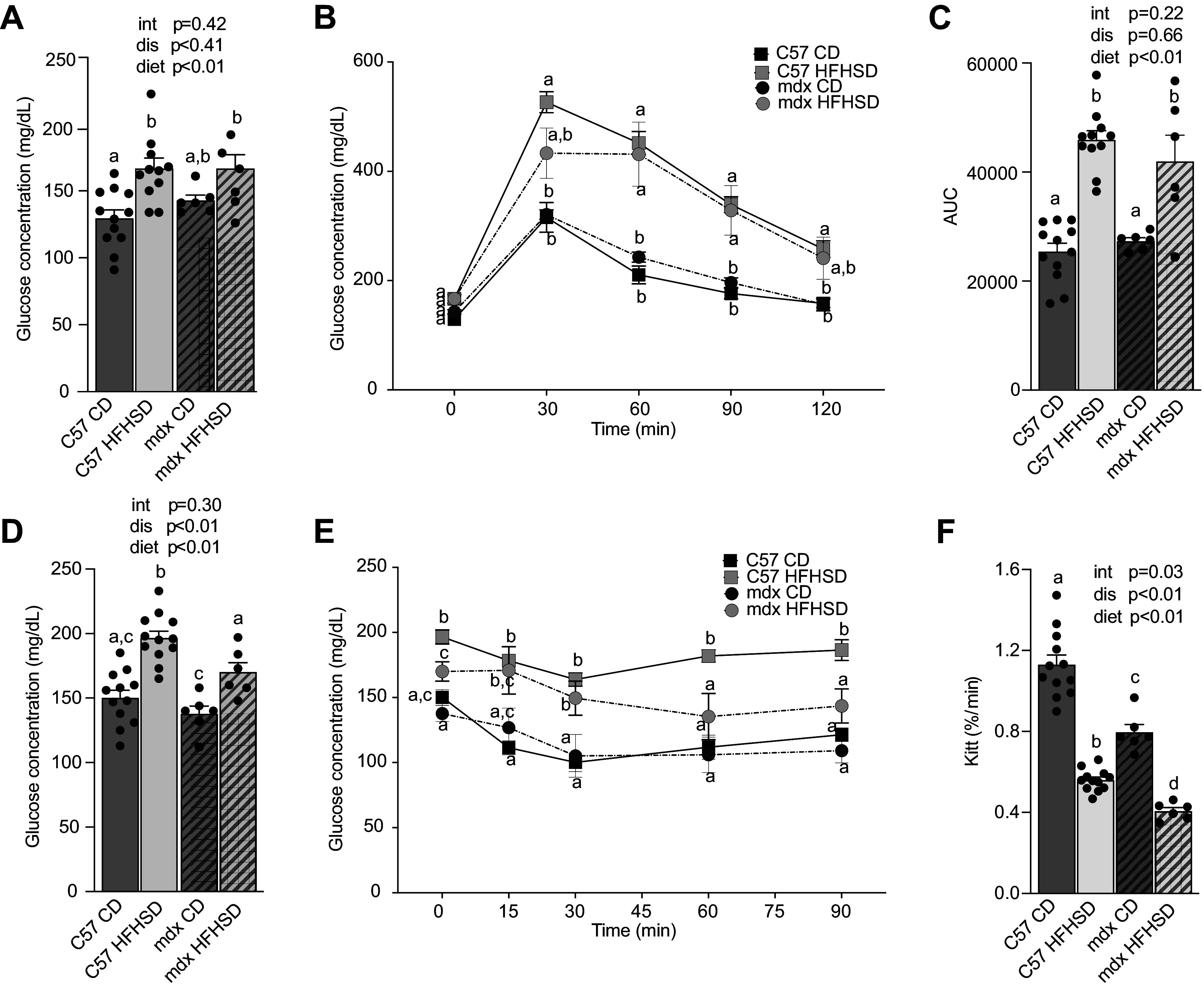
Evaluation of glucose intolerance and insulin sensitivity. Glucose and insulin tolerance tests (GTT and ITT) suggest glucose intolerance and insulin resistance in high-fat, high-sucrose diet (HFHSD)-fed groups. *A*: glucose concentrations measured after 12 h of fasting following 10 wk of diet treatment indicated hyperglycemia in HFHSD-fed groups when compared with C57 control diet (CD). *B*: In a GTT, glucose concentrations were serially measured following glucose delivery and caused higher peaks and area under curve (AUC) for HFHSD groups compared with that of CD-fed groups. C: area under curve (AUC) plotted for the GTT suggests glucose intolerance in C57-and mdx-HFHSD groups. *D*: glucose concentrations measured after 4 h of fasting following 12 wk of diet treatment indicate hyperglycemia in HFHSD groups compared with the respective CD-fed groups. *E*: glucose concentrations during an ITT indicate steeper slopes for CD-fed mice compared with HFHSD-fed mice 15–30 min after insulin injection. *F*: rate of glucose disappearance expressed as Kitt (%/min) calculated from the ITT indicates decreased insulin sensitivity in mdx- and C57 HFHSD, and mdx CD. Means ± SE is represented. Main effects (dis, disease; diet; and int, interaction) are indicated above each graph. Significance established at *P* < 0.05 for post hoc tests from two-way ANOVA and repeated-measures ANOVA. Different lowercase letters indicate differences between groups (*n* = 12 mice/group for C57 mice; *n* = 6 mice/group for mdx mice).

To determine IR, GTT and ITT were performed. After an overnight fast before GTT, basal blood glucose values were increased by 22% as a main effect of diet (*P* < 0.05; Fig. 2*A*). For basal blood glucose levels, the C57- and mdx HFHSD groups were similar and increased ∼20% (*P* < 0.05) compared with C57 CD. In the GTT, glucose delivery increased blood glucose at 30 min, and it remained elevated for 60 min in CD-fed animals and for >90 min for HFHSD-fed animals. Dystrophin deficiency did not appear to alter the rate of glucose clearance (Fig. 2*B*). When the area under the curve (AUC) was considered, there was a main effect of diet such that HFHSD-fed mice were 66% greater than CD-fed mice (*P* < 0.05) regardless of disease status, indicating the HFHSD caused glucose intolerance (Fig. 2*C*).

After a 2-h fast before ITT, blood glucose levels were increased by ∼40% as a main effect of diet (*P* < 0.05) and were increased in C57- and mdx HFHSD groups compared with the respective CD groups (*P* < 0.05; Fig. 2*D*). The Kitt was determined from the rate of glucose clearance following injection with insulin (Fig. 2*E*) and was decreased by ∼50% by the HFHSD (*P* < 0.05) in both genotypes (Fig. 2*F*). Of interest, Kitt from mdx CD was decreased ∼27% compared with C57 CD (*P* < 0.05), indicating dystrophin deficiency may contribute directly to IR. Further suppression of Kitt values in HFHSD-fed mdx mice indicates decreased insulin sensitivity.

### Tissue Mass

**Figure 3.**
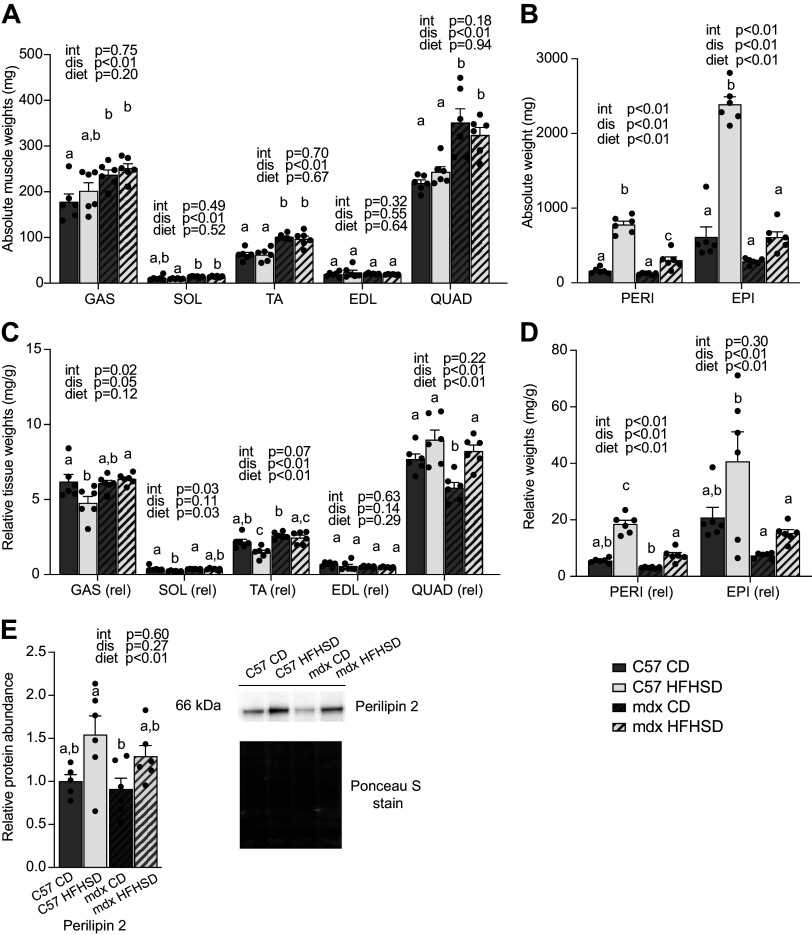
The impact of a high-fat, high-sucrose diet (HFHSD) on muscle weights and adipose deposition. *A*: dystrophin deficiency caused increased absolute muscle weights in gastrocnemius (GAS), soleus (SOL), tibialis anterior (TA), and quadriceps (QUAD). *B*: absolute adipose tissue weights from peri-renal adipose (PERI) tissue increased with HFHSD in C57 and mdx. Epididymal adipose (EPI) increased in C57 HFHSD compared with other groups. *C*: relative muscle weights (absolute weights normalized to the body weight) increased as disease main effect in GAS; increased as diet main effect in SOL; increased as disease- and decreased as diet- main effect in TA; decreased as disease main effect and increased as diet main effect in QUAD. *D*: relative adipose weights (absolute weights normalized to the body weight) increased with HFHSD in both C57 and mdx compared with respective control diet (CD) groups for PERI and increased relative EPI in C57 HFHSD compared with C57 CD. *E*: perilipin 2, a lipid droplet marker, increased as diet main effect in HFHSD groups in diaphragm whole muscle extract. Means ± SE is represented. Main effects (dis, disease; diet; and int, interaction) are indicated above each graph. Significance established at *P* < 0.05 for post hoc tests from two-way ANOVA. Different lowercase letters indicate differences between groups (*n* = 6 mice/group for all groups; identified outliers were removed according to our a priori criterion.).

There was a significant main effect of disease such that absolute gastrocnemius, soleus (SOL), tibialis anterior (TA), and quadriceps masses were larger in mdx than C57 (*P* < 0.01), whereas extensor digitorum longus (EDL) mass was similar between groups (Fig. 3). Diet did not impact absolute muscle weight. When normalized for body weight, there was a main effect of disease such that gastrocnemius and TA from mdx were larger than the corresponding muscles from C57 groups (*P* < 0.05), the mdx quadriceps were smaller than quadriceps from C57 (*P* < 0.01), and the soleus and EDL were similar between groups. The HFHSD decreased relative soleus and TA mass (*P* < 0.05) but increased relative quadriceps mass (*P* < 0.05), whereas relative gastrocnemius and EDL were similar between groups. Within C57 mice, the HFHSD decreased relative gastrocnemius mass by 9%, soleus mass by 21%, and TA mass by 17%. Within mdx mice, the HFHSD decreased relative TA mass by 14%.

Absolute perirenal adipose weights were significantly decreased by dystrophin deficiency (*P* < 0.01) and significantly increased by the HFHSD (*P* < 0.01; Fig. 3). The HFHSD increased perirenal adipose mass in C57 mice by ∼3.85-fold but only by 1.5-fold in mdx mice compared with CD-fed C57 and mdx mice, respectively, which contributed to a significant interaction. When perirenal adipose was normalized to body weight, there were main effects of diet and disease such that C57 was greater than mdx and HFHSD was greater than CD. The HFHSD increased relative perirenal adipose in C57 mice by ∼2.3-fold and in mdx mice by ∼1.4-fold compared with corresponding CD-treated groups, contributing to a significant interaction. Similar, though not identical, outcomes were discovered in the epididymal fat pad. In addition, perilipin 2, an intramuscular lipid droplet marker, was increased in diaphragms from HFHSD-fed mice (Fig. 3).

### Lipidomics

**Figure 4.**
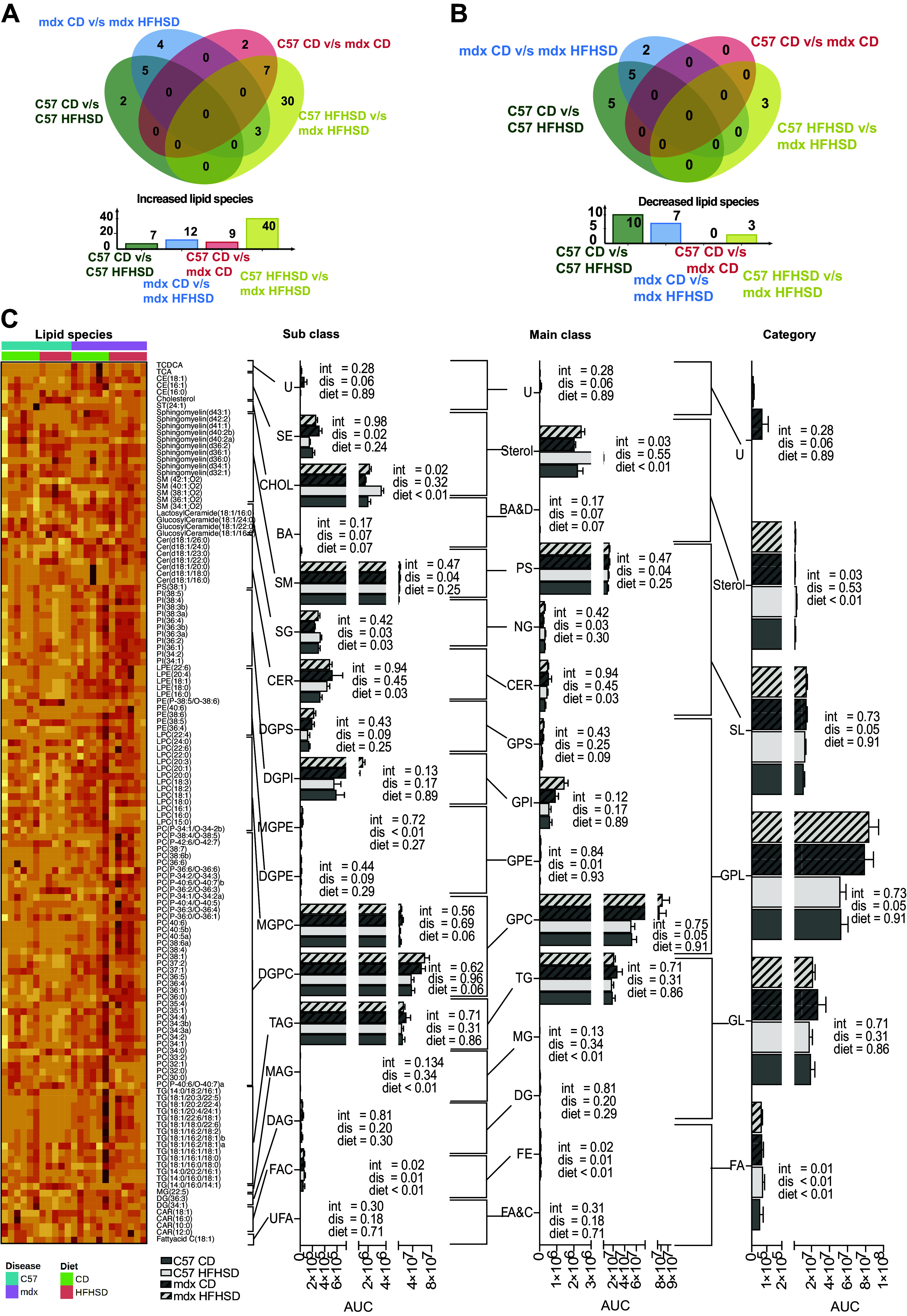
Serum lipidomic analyses indicated common and unique consequences of disease and diet. *A*: Venn diagrams for upregulated lipid species in the four pairwise comparisons used, including C57 control diet (CD) with C57 high-fat, high-sucrose diet (HFHSD), mdx CD with mdx HFHSD, C57 CD with mdx CD, and C57 HFHSD with mdx HFHSD. As an example, data are presented such that mdx CD vs. mdx HFHSD indicates increase in mdx HFHSD compared with mdx CD. *B*: Venn diagrams for downregulated lipid species in the four pairwise comparisons used, including C57 CD with C57 HFHSD, mdx CD with mdx HFHSD, C57 CD with mdx CD, and C57 HFHSD with mdx HFHSD. *C*: heatmap and classification of the lipid species. The heatmap gradient is ranged from light yellow (low abundance) to bright red (high abundance). The lipid species are classified into different subclasses, which are further classified into main classes, and the main classes are classified into major lipid categories. For the subclasses, main classes, and categories, means ± SE is represented. Main effects (dis, disease; diet; and int, interaction) are indicated next to each graph. Significance established at *P* < 0.05 for post hoc tests from two-way ANOVA [C57 CD *n* = 5 (missing sample); *n* = 6/group for remaining groups]. The subclass group includes U, uncategorized; SE, steryl esters; CHOL, cholesterol and derivatives; BA, C24 bile acids; SM, sphigomyelins (ceramidephosphocholines); SG, Simple Glc series; CER, ceramides (*N*-acylsphingosines); DGPI, diacylglycerophosphoinositols; MGPE, monoacylglycerophosphoethanolamines; DGPE, diacylglycerophosphoethanolamines; MGPC, monoacylglycerophosphocholines; DGPC, diacylglycerophosphocholines; TAG, triacylglycerols; MAG, monoacylglycerols; DAG, diacylglycerols; FAC, fatty acyl carnitines; and UFA, unsaturated fatty acids. The main class group includes U, uncategorized sterols; BAD, bile acids and derivatives; PS, phosphosphingolipids; NG, neutralglycosphingolipids; CER, ceramides; GPS, glycerophosphoserines; GPI, glycophosphoinositols; GPE, glycerophosphoethanolamines; GPC, glycerophosphocholines; TG, triradylglycerols; MG, monoradylglycerols; DG, diradylglycerols; FE, fatty esters; FA&C, fatty acid and conjugates. The category group includes U, uncategorized sterols; SL, sphingolipids; GPL, glycerophospholipids; GL, glycerolipids; and FA, fatty acyls.

We performed lipidomics to assess the alterations in circulating lipids caused by DMD and/or HFHSD. Differential expressions of individual lipid species in each comparison of interest were demonstrated in volcano plots and Venn diagrams (Supplemental Fig. S1, *A–D*). HFHSD increased seven and decreased 10 lipid species (*q*-value cut off = 0.1) in C57 HFHSD compared with C57 CD and increased 12 and decreased seven lipid species (*q*-value cut off = 0.1) in mdx HFHSD compared with mdx CD (Fig. 4*A*). Interestingly, when the genotype effect was considered, no lipid species were significantly decreased in mdx CD compared with C57 CD (Fig. 4*B*), but 9 lipid species were increased in mdx CD compared with C57 CD (Fig. 4*A*). When the C57 HFHSD was compared with mdx HFHSD, 40 lipid species were increased and 3 were decreased in mdx HFHSD compared with C57 HFHSD (Fig. 4, *A* and *B*, and Supplemental Fig. S1, *A*–*D*).

Furthermore, the Venn diagrams were also used to elucidate the common and unique signatures of the lipid species (Fig. 4*A*). Five lipid species were commonly increased with HFHSD in mdx and C57 mice (intersection of the comparisons: C57 CD vs. C57 HFHSD and mdx CD vs. mdx HFHSD), seven were commonly increased with disease regardless of diet (intersection of the comparisons C57 CD vs. mdx CD and C57 HFHSD vs. mdx HFHSD), and five were commonly decreased with disease (intersection of the comparisons C57 CD vs. mdx CD and C57 HFHSD vs. mdx HFHSD). These individual lipid species are identified in Supplemental Table S1.

The uniquely expressed lipid species include four increased in mdx HFHSD compared with mdx CD, two increased in C57 HFHSD compared with C57 CD, two upregulated in mdx CD compared with C57 CD, and 30 upregulated in mdx HFHSD compared with C57 HFHSD. There were two uniquely downregulated lipid species in mdx CD versus mdx HFHSD [lysophosphatidylcholine (LPC) 15:0 and phosphatidylcholine (PC) 32:0], five in C57 CD versus C57 HFHSD [C24 Sphingomyelin (18:1/24:0), PC 37:2, PC 35:1, LPE 16:0, and mono(acyl|alkyl)glycerols (MG) 22:5], and three in C57 HFHSD versus mdx HFHSD (Cholesterol, PC P-36:2/O-36:3, and Sphingomyelin d40:2a). In addition, the number of differentially expressed lipid species in the C57 HFHSD versus mdx HFHSD comparison (40 lipid species increased and 3 lipid species decreased; Fig. 4, *A* and *B*) suggests the possibility of disease contributing to lipid accumulation in an HFHSD environment.

The 133 identified lipid species were classified into 18 subclasses, 15 main classes, and 6 categories (Fig. 4*C*). Among the subclasses, fatty acylcarnitines (FAC) were elevated with disease (*P* = 0.0104) and diet (*P* = 0.0037) as the main effects, and there was an interaction (*P* = 0.0171). The effect of the disease was evident with increased sphingomyelins (ceramide phosphocholines, *P* = 0.0398), steryl esters (SE, *P* = 0.024), monoacyl glycerophosphoethanolamines (MGPE, *P* = 0.0054), and simple Glc series (SG, *P* = 0.0256) as a main effect of the disease. There was an increased main effect of diet with cholesterol and derivatives (CHOL, *P* < 0.0001), monoacyl glycerols (MAG, *P* = 0.0021), and ceramides (*N*-acylsphingosines, CER, *P* = 0.0314). The CHOL also had interaction main effects (*P* = 0.0218) as mdx HFHSD was higher than C57 HFHSD.

A further categorization of the lipid species into main classes indicated glycerophosphocholines (GPC) constitute the majority of the lipid main classes in all four groups (Fig. 4*C*). Among the main classes, fatty esters (FE) had disease (*P* = 0.0104), and diet (*P* = 0.0037) main effects such that fatty esters (FE) increased with disease and HFHSD, and an interaction (*P* = 0.0171) main effect such that suppression of diet effect by disease in mdx HFHSD. Ceramides (CER, *P* = 0.0314), sterols (*P* = 0.0003), and monoradylglycerols (MG, *P* = 0.0021) were increased as a main effect of diet. Glycerophosphocholines (GPC, *P* = 0.0523), glycerophosphoethanolamines (GPE, *P* = 0.0114), neutral glycosphingolipids (NG, *P* = 0.0256), and phosphosphingolipids (PS, *P* = 0.0398) were increased in mdx compared with C57 as a main effect of disease.

Among the categories, glycerophospholipids (GPL) constituted the majority of plasma lipid species in the four treatment groups (Fig. 4*C*). Fatty acyls (FA) were impacted by a main effect of disease (*P* = 0.0094), diet (*P* = 0.0037), and interaction (*P* = 0.016) such that fatty acyls were increased with HFHSD and DMD. Glycerophospholipids (GPL, *P* = 0.052) and sphingolipids (SL, *P* = 0.0396) were also increased as a main effect of the disease. Sterol lipids (Sterols) were increased as a main effect of diet (*P* = 0.0314) and had a significant interaction (*P* = 0.0314) such that a suppression of diet effect by disease was observed in mdx HFHSD.

### Muscle Injury

**Figure 5.**
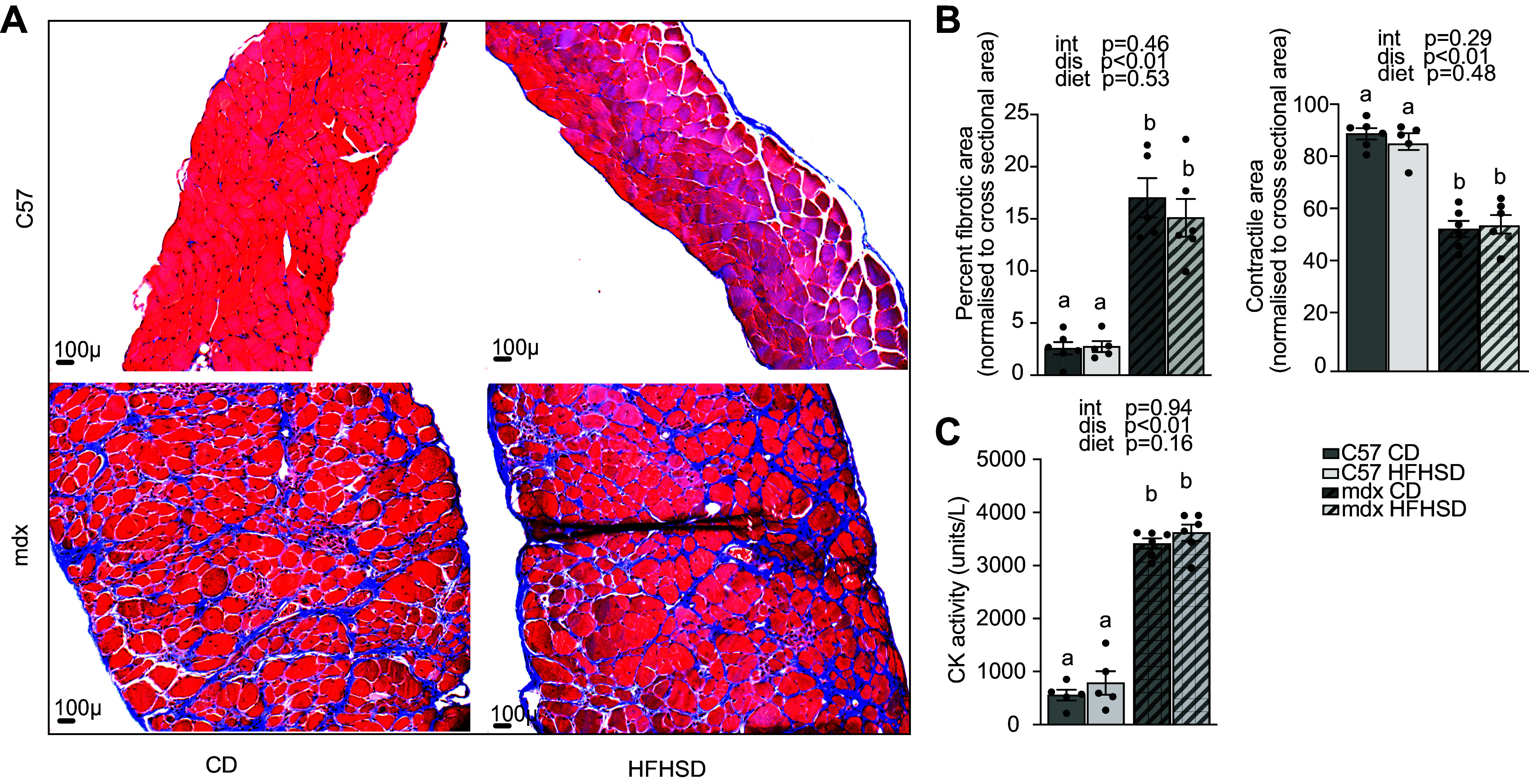
Assessment of muscle injury. *A*: representative ×20 images of diaphragm sections that are stained by Masson’s trichrome staining showing cytoplasm (red), collagen (blue), and nuclei (dark brown) [C57 high-fat, high-sucrose diet (HFHSD) *n* = 5 (missing sample); *n* = 6/group for remaining groups]. *B*: dystrophin deficiency increased percent fibrotic area (blue-stained area expressed relative to the total cross-sectional area) and decreased percent contractile area (red-stained area expressed relative to the total cross-sectional area) as a main effect of disease. *C*: serum creatine kinase activity was increased as a main effect of disease [C57control diet (CD) *n* = 5 (missing sample); *n* = 6/group for remaining groups]. Main effects (dis, disease; diet; and int, interaction] are indicated above each graph. Significance established at *P* < 0.05 for post hoc tests from two-way ANOVA. Different letters indicate differences between groups.

Diaphragm cross-sections were used for trichrome staining to objectively quantify histopathological changes (Fig. 5). Predictably, in the trichrome-stained sections, fibrosis was increased as a main effect of disease; however, it was not impacted by diet (Fig. 5*B*). Likewise, the contractile area from trichrome staining was decreased as a main effect of disease and was not impacted by diet (Fig. 5*C*). Finally, serum creatine kinase activity was significantly elevated by disease (*P* < 0.0001) but not diet (*P* = 0.1609) (Fig. 5*D*).

### Cellular Dysfunction

**Figure 6.**
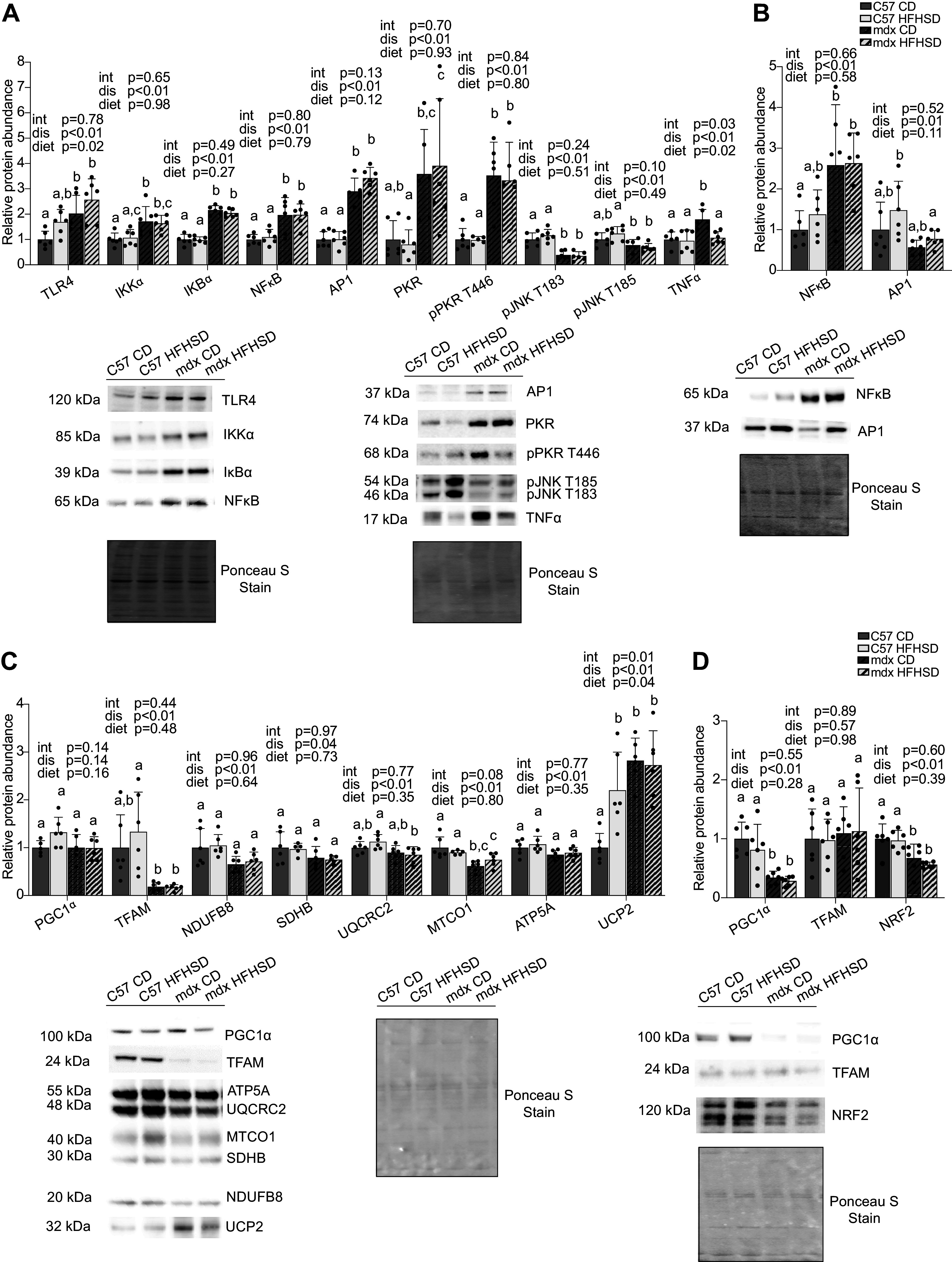
Markers of inflammatory signaling and mitochondrial abundance were altered by disease. *A*: in whole muscle extract, all inflammatory signaling markers except pJNK T183 and pJNK T185 had an increased main effect of disease in mdx diaphragm compared with C57. Toll-like receptor (TLR4) was increased, and TNFα decreased as a main effect of diet with high-fat, high-sucrose diet (HFHSD). *B*: in the nuclear fraction, NF-κB was increased and AP1 decreased as a main effect of disease in mdx diaphragm compared with C57. *C*: in whole muscle extract, all markers of mitochondrial biogenesis and oxidative phosphorylation except peroxisome proliferator-activated receptor gamma coactivator 1-α (PGC1α) and uncoupling protein 2 (UCP2) were reduced as a main effect of disease in mdx diaphragm compared with C57. UCP2 had disease, diet, and interaction main effects, whereas PGC1α was similar between groups. *D*: in the nuclear fraction, PGC1α and NRF2 were decreased as a main effect of disease. For all panels, representative sample blots and Ponceau S stains are included. Main effects (dis, disease; diet; and int, interaction) are indicated above each graph. Significance established at *P* < 0.05 for post hoc tests from two-way ANOVA. Different lowercase letters indicate differences between groups (*n* = 6/group for all groups; identified outliers were removed according to our a priori criterion.

Whole muscle extract from the diaphragm was used to probe pathways that may be altered by disease, diet, or the interaction of disease and diet. Dystrophin deficiency and IR were previously found to independently alter inflammatory signaling in muscle (47, 48). We discovered that Toll-like receptor (TLR4), a cellular pattern recognition receptor, was increased by 70% (*P* = 0.0013) as a main effect of disease and ∼2.5-fold by diet (*P* = 0.0242; Fig. 6*A*). Inhibitor of nuclear factor κB kinase subunit α (IKKα), nuclear factor of κ light polypeptide gene enhancer in B-cells inhibitor α (IKBα), nuclear factor κ-light-chain-enhancer of activated B cells (NF-κB), activator protein 1 (AP1), protein kinase R (PKR), and PKR phosphorylated at T446 (pPKR T446) were increased by 1.5- to 4-fold (*P* < 0.05) in mdx compared with C57 but were not altered by diet (Fig. 6*A*). pJNK T183 (*P* = 0.0006) and pJNK T185 (*P* = 0.0001) were decreased by ∼30% and 60%, respectively, as a main effect of disease but not diet. TNFα, an inflammatory cytokine, was increased in whole homogenate in mdx compared with C57 (*P* = 0.0058) and decreased with diet in HFHSD compared with CD (*P* = 0.0152), with a significant interaction (*P* = 0.0326). Of note, although TNFα was similar between CD and HFHSD in C57, it was decreased by ∼40% (*P* = 0.0094) in mdx HFHSD. Since TNFα was higher in mdx CD than in all other groups (*P* < 0.05), there is a possibility that the HFHSD diet suppressed the TNFα expression in mdx mice. Since the nuclear translocation of transcription factors such as NF-κB and AP1 are required for the activation of downstream genes, these proteins were probed for their abundance in a nuclear fraction. NF-κB was increased in the dystrophic diaphragm compared with C57 by approximately twofold (*P* = 0.0011). AP1 was decreased by ∼50% (*P* = 0.0125) in mdx compared with C57 as a main effect of disease but not diet.

Furthermore, we considered how mitochondrial abundance could be altered by disease and HFHSD. Total PGC1α was similar between groups in whole muscle extract (Fig. 6*C*); however, in a nuclear fraction, peroxisome proliferator-activated receptor gamma coactivator 1-α (PGC1α) was decreased by 65% (*P* < 0.0001) and nuclear factor-like 2 (NRF-2) was decreased by 40% as a function of disease (*P* = 0.0002; Fig. 6*D*). Dystrophin deficiency decreased transcription factor A, mitochondrial (TFAM) by ∼80% (*P* = 0.0002) in whole homogenates, though was similar between groups in nuclear fractions. We discovered that the mitochondrial markers, NADH:ubiquinone oxidoreductase subunit B8 (NDUFB8), succinate dehydrogenase complex iron sulfur subunit B (SDHB), ubiquinol-cytochrome *c* reductase core protein 2 (UQCRC2), mitochondrially encoded cytochrome *c* oxidase I (MTCO1), and ATP synthase F1 subunit α (ATP5A), were decreased by ∼15 to 35% (*P* = 0.0004 to *P* = 0.0387) with disease but not diet (Fig. 6*C*). Uncoupling protein 2 (UCP2), a protein that uncouples oxidative phosphorylation from ATP synthesis, was increased by ∼1.5-fold by disease (*P* = 0.0001) and diet (*P* = 0.0370), though the change in diet was caused by the C57 HFHSD group, which produced an interaction (*P* = 0.0160, Fig. 6*C*). Furthermore, UCP2 was higher in C57 HFHSD (2.2-fold, *P* = 0.0127), mdx CD (2.8-fold, *P* = 0.0002), and mdx HFHSD (2.7-fold, *P* = 0.0004) groups compared with C57 CD (Fig. 6*C*).

**Figure 7.**
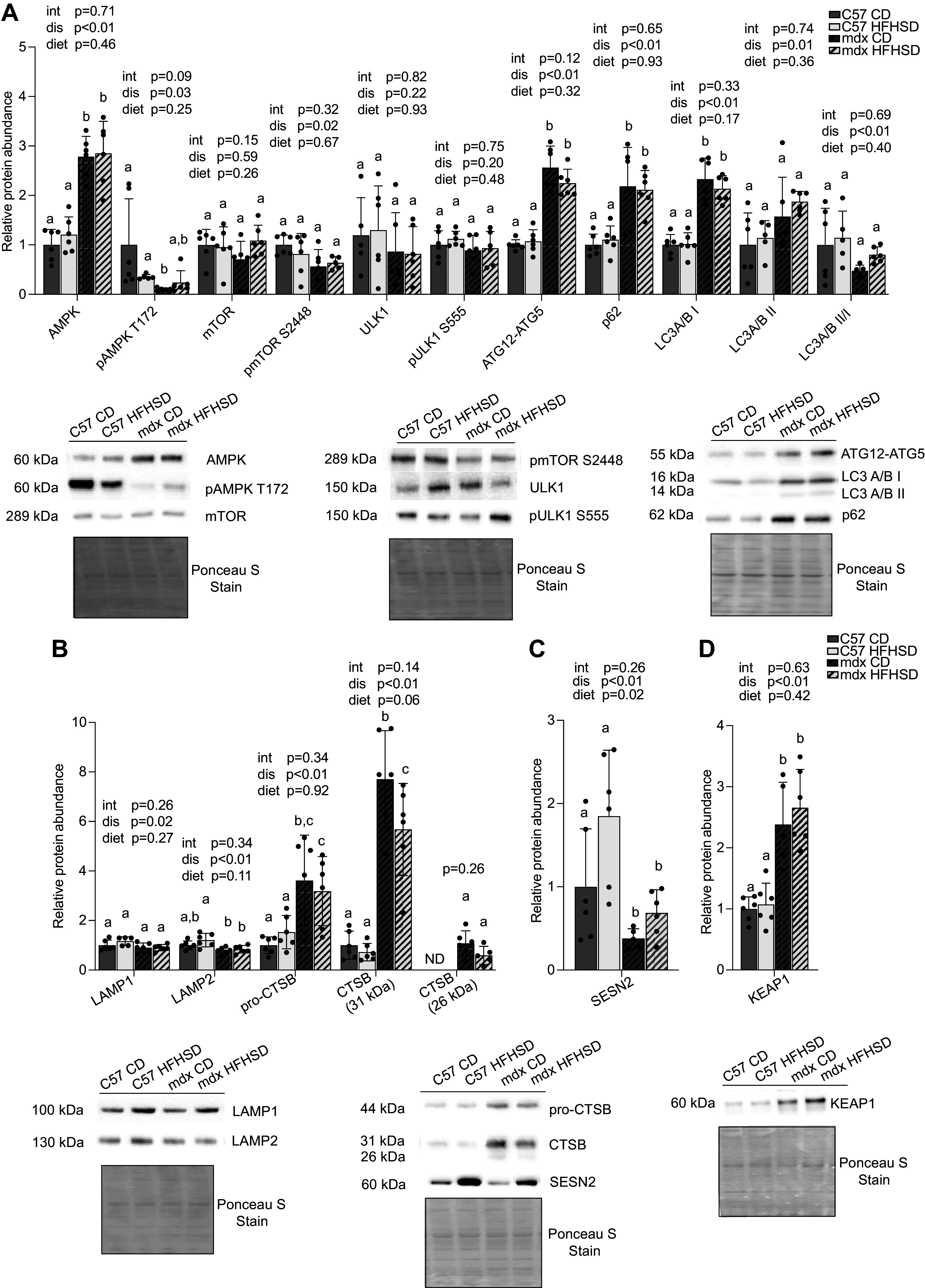
Assessment of autophagy and lysosomal markers cell signaling using Western blots. *A*: in whole muscle extract, AMPK, ATG12-ATG5, p62, LC3A/B I, and LC3A/B II were increased as a main effect of disease in mdx diaphragm compared with C57. pAMPK T172, pmTOR S2448, and LC3A/B II/I were decreased as a disease main effect in mdx diaphragm compared with C57. *B*: in whole muscle extract, lysosomal markers LAMP1 and LAMP2 were decreased, and several different forms of lysosome function marker cathepsin B (CTSB) were increased as a main effect of disease in dystrophic diaphragms compared with C57. Note, the 26-kDa CTSB band was not detected (ND) in muscle from C57 mice. *C*: Sestrin2 (SESN2) was decreased by disease and increased by diet main effects in diaphragm from mdx when compared with C57. *D*: KEAP1 in nuclear fraction indicates nuclear accumulation with disease. For all panels, representative sample blots and Ponceau S stains are included. Main effects (dis, disease; diet; and int, interaction) are indicated above each graph. Significance established at *P* < 0.05 for post hoc tests from two-way ANOVA. Different lowercase letters indicate differences between groups (*n* = 6 mice/group for all groups; identified outliers were removed according to our a priori criterion).

Autophagy has been previously found to be altered by dystrophin deficiency as well as insulin resistance (3, 5, 49–52). Overall, autophagy markers were altered by disease but not diet (Fig. 7). AMP-activated protein kinase (AMPK) was increased by 2.8-fold (*P* < 0.0001) in mdx mice compared with C57 as a main effect of disease (Fig. 7*A*); however, pAMPK T172 was decreased by ∼70% (*P* = 0.0319) in mdx mice compared with C57. Mechanistic target of rapamycin kinase (mTOR) phosphorylated at S2448 (pmTOR S2448) was decreased by ∼20% (*P* < 0.0247) as a main effect of the disease. The autophagosome formation markers, ATG12-ATG5 (∼2-fold, *P* < 0.0001) and microtubule-associated protein 1 light chain 3 A/B (LC3A/B) I (2.23-fold, *P* < 0.0001) were increased with disease. LC3A/B II, a marker of autophagosome abundance, was increased ∼1.5-fold (*P* = 0.0119), and p62, an inverse correlate of autophagosome degradation, was increased ∼2-fold (*P* < 0.0001), in mdx compared with C57 but were not altered by diet.

Lysosomal markers lysosomal-associated membrane protein 1 (LAMP1) and lysosomal-associated membrane protein 2 (LAMP2) were decreased by 20% (*P* = 0.0231) and 27% (*P* < 0.0001), respectively, as a main effect of the disease. pro-CTSB was 2.68-fold higher (*P* = 0.0003) in mdx compared with C57 and the mature cathepsin B (CTSB) (31 kDa) was approximately sevenfold higher in mdx compared with C57 regardless of dietary treatment (*P* < 0.0001, Fig. 7*C*). The 26-kDa mature cathepsin B (CTSB) band was detected only in the mdx CD and mdx HFHSD groups and they were similar to each other (Fig. 7*C*). Of interest, the mdx HFHSD had ∼30% less (*P* < 0.05) CTSB (31 kDa) compared with mdx CD. Sestrin2 (SESN2), a protein involved in regulating stress responses including inflammation, oxidative stress, endoplasmic reticulum stress, and autophagy, and also known to attenuate metabolic defects due to IR, was also evaluated (53, 54). Relative abundance of SESN2 was decreased by ∼60% as a main effect of disease (*P* = 0.0012) but increased by 1.8-fold as a main effect of diet (*P* = 0.0237; Fig. 7*C*). In addition to NRF2 (a transcription factor critical for SESN2 expression), SESN2 is also regulated by the release of Kelch-like ECH-associated protein 1 (KEAP1) (NRF2 suppressor) interaction with NRF2 in the cytoplasm. Surprisingly, there was a nuclear accumulation of KEAP1 (increased by 2.4-fold, *P* < 0.0001) in mdx mice as a main effect of the disease.

### Metabolomics

**Figure 8.**
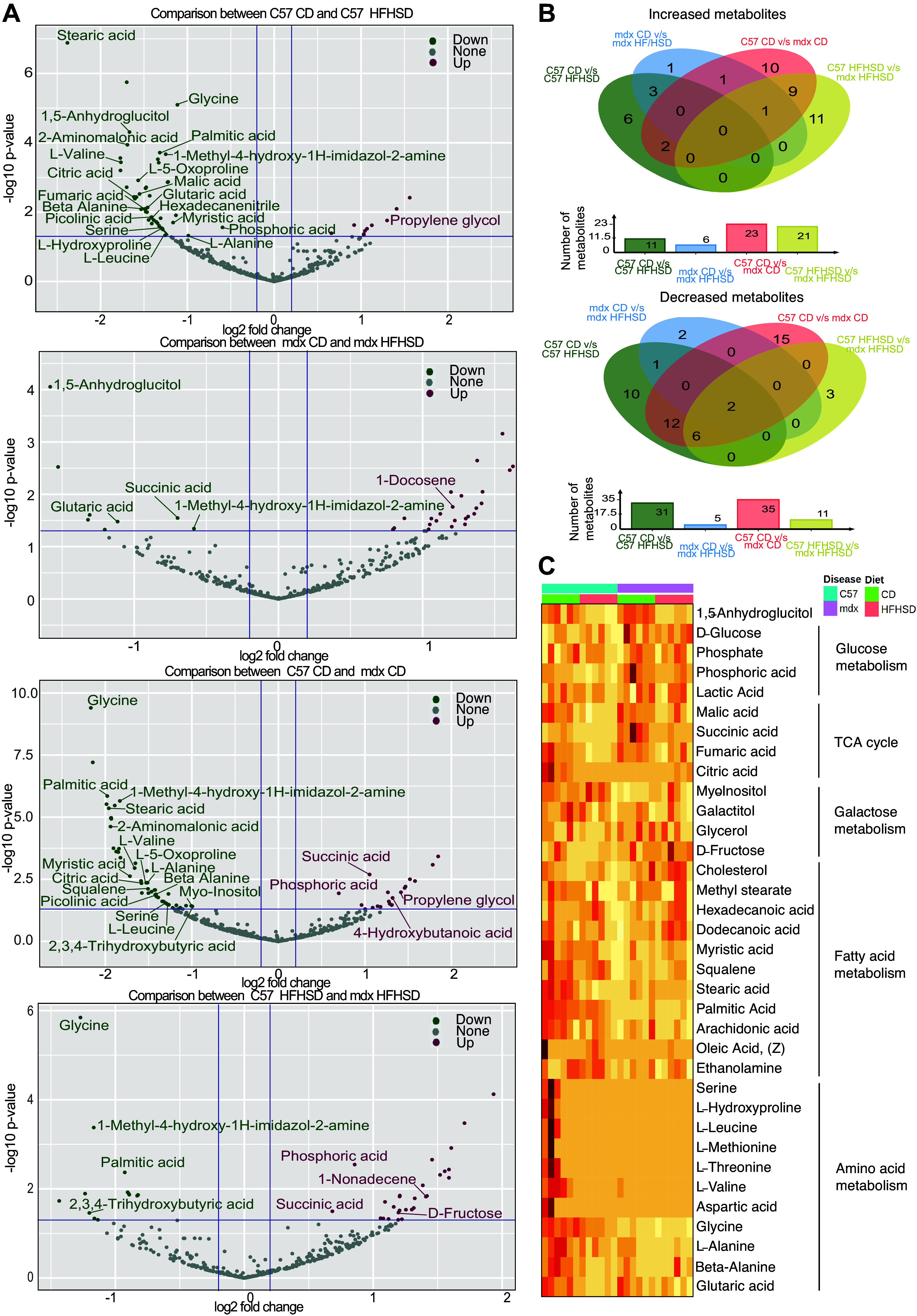
Metabolomic analyses indicated common and unique metabolic signatures for disease and diet. As an example, data are presented such that mdx control diet (CD) vs. mdx high-fat, high-sucrose diet (HFHSD) indicates increased in mdx HFHSD compared with mdx CD. *A*: volcano plots showing features that are increased and decreased in *1*) C57 HFHSD when compared with C57 CD, *2*) mdx HFHSD when compared with mdx CD, *3*) mdx CD when compared with C57 CD, and *4*) mdx HFHSD when compared with C57 HFHSD. Significance established at *P* < 0.05 and log2 fold change (FC) = 0.1. *B*: Venn diagrams for upregulated and downregulated metabolites in the four pairwise comparisons used including C57 CD with C57 HFHSD, mdx CD with mdx HFHSD, C57 CD with mdx CD, and C57 HFHSD with mdx HFHSD. *C*: a heat map was constructed by grouping detected and identified metabolites into major metabolic pathways. (*n* = 6 mice/group for all groups). The heatmap gradient is ranged from light yellow (low abundance) to bright red (high abundance).

As a preliminary step in identifying the extent to which O/IR alters muscle metabolism in dystrophic skeletal muscle, we performed a nontargeted metabolomics experiment using quadriceps muscle. In this analysis, 592 metabolites were detected, and 72 were identified. The differentially expressed metabolites in each comparison are represented in volcano plots (Fig. 8*A*). Of the detected metabolites, 11 were increased and 31 were decreased in C57 HFHSD compared with C57 CD [*P* < 0.05, log_2_ fold change (FC) > 0.1] (Fig. 8*B*). In mdx CD, when compared with C57 CD, 23 metabolites were increased and 35 were decreased (*P* < 0.05, log_2_FC > 0.1). When mdx HFHSD was compared with mdx CD, six metabolites were increased and five metabolites were decreased (*P* < 0.05, log_2_FC > 0.1). Among the decreased identified metabolites, 1,5-anhydroglucitol [a biomarker of hyperglycemia (55)], 1-methyl-4-hydroxy-1*H*-imidazol-2-amine, and glycine were common for both C57- and mdx-HFHSD compared with corresponding CD-fed mice. In the mdx HFHSD group, two metabolites were uniquely decreased [succinic acid, a tricarboxylic acid cycle (TCA) intermediate, was identified out of the two], and 1-docosene was uniquely increased compared with all other groups. HFHSD feeding in C57 differentially regulated 36 metabolites (8 increased and 28 decreased) in C57 HFHSD but not in mdx HFHSD compared with their respective controls. Interestingly, 20 metabolites were differentially regulated in the C57 CD compared with mdx CD suggesting some differences may be driven by disease. A two-way ANOVA (Supplemental Fig. S2) indicated differences between groups, treatments, and interactions. Pathway enrichment analysis using the significant features identified from this comparison (Supplemental Fig. S2), suggested changes associated with pathways including glutathione and glutamate metabolism, amino acid metabolism [including branched-chain amino acid (BCAA) metabolism], and fatty acid metabolism (Fig. 8*C*). Of interest, location enrichment indicated subcellular localization of some metabolites in mitochondria (Supplemental Fig. S2).

## DISCUSSION

Duchenne muscular dystrophy is often associated with metabolic complications such as IR, metabolic syndrome, and diabetes in boys/men with DMD (14, 15, 56, 57) making nutritional management a critical component in the care of those with DMD (18, 20, 23). However, despite this frequency, the extent to which diet-induced IR (DI-IR) may promote disease progression and severity is unknown. Cellular dysfunctions associated with IR are independent of and overlap with dysfunctions caused by dystrophin deficiency, providing substantial rationale to suspect additive or even synergistic impacts. Furthermore, O/IR in mdx mice may allow this model to better recapitulate DMD by intensifying the generally mild disease phenotype and/or rate of disease progression. Therefore, the purpose of this investigation was to understand the impact of O/IR induced by HFHSD-feeding on dystrophic pathophysiology.

In this investigation, we used an HFHSD to induce O/IR in mdx mice. Predictably, this diet increased body weight and adiposity and caused hyperglycemia, glucose intolerance, and IR in C57 mice. This study is the first report, to our knowledge, of diet-induced hyperglycemia, glucose intolerance, and IR in mdx mice. In this investigation, mdx CD mice were also insulin resistant. This finding is in good agreement with previous demonstrations of IR in boys with DMD, even in those who are underweight (15), and adds to a growing body of literature suggesting that dystrophin deficiency may promote IR (12). Interestingly, this also supports the role of the DGC as a regulator of insulin receptor signaling (12). However, caution should be taken when interpreting these data as caloric consumption was similar between C57 HFHSD, mdx CD, and mdx HFHSD groups. Hence, our finding of IR in the mdx CD group could be alternatively interpreted to suggest that the overconsumed calories in mdx CD may drive IR. Previous studies indicate that dystropathology in mdx mice may result in increased metabolic demands (12, 29); hence, increased caloric consumption in mdx mice may be a mechanism to meet these demands. Indeed, mdx mice had much lower adiposity when compared with C57 mice within dietary treatments. Furthermore, given the differences in outcomes (fat pad weight, insulin sensitivity, and glycemia) despite similar caloric consumption between mdx CD and mdx HFHSD, these data also indicate that dietary composition, not just caloric consumption, impacts obesity-related measures within the context of dystrophinopathy. Estimates of metabolic inefficiency were beyond the scope of this investigation, but Radley-Crabb et al. (12) have previously reported increased energy expenditure in mdx mice.

Metabolic alterations in DMD as well as IR may result in abnormal plasma lipid levels leading to dyslipidemia (12, 29, 58). In concordance with previous reports, we found elevations in glycerophospholipids with disease in dystrophic mice (59) and triacylglycerol (TAGs) were similar between groups (29). The elevations in ceramides, fatty acylcarnitines, cholesterol and derivatives, and monoacyl glycerols at the subclass level, are suggestive of lipotoxicity with HFHSD feeding in dystrophic mice (39, 60). Though the sterol main class (constituting both cholesterol and derivatives and steryl esters) was similar between mdx groups as previously reported (29), further consideration of this class indicated increased steryl esters but not in cholesterol and derivatives. The elevation in sphingolipids and glycerophospholipids in mdx mice, which are important components of the cell membrane and cell processes such as inflammation, are in good agreement with the previous findings of increased muscle damage and inflammatory signaling in mdx mice (58, 61). In addition, the significant disease-diet interactions with fatty acyls and sterol lipids are another indication of the impact that DI-IR can have on DMD. These results clearly indicate an accumulation of plasma lipids in HFHSD-fed C57 and mdx mice. The lipidomic data also suggest common and unique lipidomic consequences of HFHSD-induced IR within the context of DMD.

Predictably, dystrophin deficiency resulted in histological muscle injury; however, and counter to our hypothesis, muscle injury was not potentiated by an HFHSD in mdx mice. This finding contradicts previous work where a 16% fat diet decreased muscle injury (28), though we note substantial differences between diets in these investigations. The outcomes described herein are in good agreement with a previous study using a Western diet (0.2% total cholesterol, 21% fat, and 34% sucrose) in that dystrophic muscle injury and serum CK were altered as a function of disease but not diet (29). Although the previous work demonstrated dyslipidemia when dystrophic, ApoE knockout mice were fed this diet, IR was not considered, though some parameters of muscle injury were increased in gastrocnemius and tibialis brachii muscles but not diaphragm, quadriceps femoris, and cardiac muscles (29). Collectively, these outcomes suggest that overt muscle injury in the diaphragm, the muscle that most closely recapitulates human disease progression (62), is not driven or promoted by an HFHSD, however, how function or metabolism may be altered is unknown.

The responses in dystrophin deficiency or IR in skeletal muscle include some common physiological mediators. Inflammatory signaling is frequently altered with DMD and IR. Our finding of increased TLR4 expression with both dystrophin deficiency as well as HFHSD groups support previous reports (47, 48, 63), and is suggestive of additive effects of DI-IR on dystrophin deficiency. TNFα was previously found to be increased with disease (64), and in good congruence, we found increased TNFα as a main effect of the disease; however, a reduction in TNFα as a main effect of diet was contradictory to published reports (65). In total, disease-mediated inflammatory signaling is in good agreement with previous reports in mdx mice and in patients with DMD (4, 64, 66–69). The HFHSD did not increase inflammatory signaling in muscle from C57 mice, however, the literature is equivocal regarding this outcome (47, 65, 70). Notably, aside from the aforementioned change in TLR4 expression, the HFHSD failed to promote inflammatory signaling in dystrophic muscle.

Mitochondrial dysfunction is associated with dystrophin deficiency as well as IR (71–73) and may disrupt energy homeostasis, ATP production, and redox balance among other roles. In this investigation, dystrophin deficiency caused a reduction in markers of mitochondrial abundance, which is consistent with previous findings (7, 74) and may be driven, at least in part, by the decreased nuclear abundance of transcription factors that promote mitochondrial biogenesis including PGC1α and NRF2 (75, 76). Conversely, an HFHSD did not alter markers of mitochondrial abundance or localization of related transcription factors. Interestingly, UCP2, a protein that uncouples oxidative phosphorylation from ATP synthesis, was increased as a main effect of disease and by diet (largely in C57 mice). UCP2 participates in the regulation of energy metabolism and redox balance (77, 78). Increased UCP2 in O/IR mice may serve as a mechanism to introduce metabolic inefficiency and limit nutrient excess. In a disease like DMD, where there is an apparent mitochondrial deficiency, decreased mitochondrial function, and an ATP insufficiency, it is unclear what advantage increased UCP2 provides. We also note that despite a diet-induced elevation in UCP2, there was not an additive effect in the HFHSD-mdx mice raising the possibility of a biological ceiling. Given its role as an uncoupler of the electron transport chain, it is reasonable to suggest that UCPs contribute to systemic metabolic inefficiency in mdx mice and therefore to decreased adiposity; however, how this function interacts with a HFHSD is unclear as is the robust excess adiposity observed in HFHSD-C57 mice despite increased UCP2 expression.

In a similar fashion to inflammatory signaling, upstream activation of autophagy and degradation of autophagosomes were affected by disease but not DI-IR. Decreased pAMPK T172 (autophagy activation marker) and decreased pmTOR S2448, with increased ATG12-ATG5 (autophagosome formation marker) in dystrophic mice compared with healthy mice were in concordance with previous findings. Consistent with previous work in diaphragms, we discovered increased p62 and LC3 II with disease, suggestive of blunted degradation and accumulation of autophagosomes (5, 49, 79). Consideration of autophagosome escape was beyond the scope of this investigation (49). Similar, but not identical to, our previous finding in diaphragm muscles from D2-mdx, a possible compensatory mechanism for decreased lysosomal abundance by an increase in mature CTSB was found in dystrophic muscles (5). Although impaired autophagy was previously reported with DI-IR (51, 80), there are also reports of maintained degradation of autophagosomes with diet-induced obesity and autophagy remaining responsive during IR (81–83).

SESN2 is an antioxidant protein that acts as a cellular signaling hub for multiple cellular processes including, but not limited to, autophagy, inflammatory signaling, oxidative stress response, endoplasmic reticulum stress, and the unfolded protein response (53, 84–87). We identified a differential response of SESN2 to dystrophin deficiency and HFHSD, suggesting that SESN2 is sensitive to the cellular and metabolic effects of both DMD and DI-IR. In good agreement with our findings, SESN2 expression was increased during hypernutrition in the liver and muscles and maintained metabolic homeostasis in obese livers so as to attenuate the systemic effects of IR, whereas ablation of SESN2 increased obesity-induced glucose intolerance, IR, and hepatic steatosis (53). Indeed, disease-mediated reductions in SESN2 may promote IR in dystrophic skeletal muscle as observed herein, even in CD-fed mdx mice. Antithetical expression of SESN2 by diet and disease is suggestive of independent mechanisms regulating SESN2 abundance during IR and dystrophin deficiency and is also reflective of the complexity of SESN2 regulation. For example, nuclear localization of NRF2 [a transcription factor that regulates (88) and is regulated by (85) SESN2], was decreased with disease, which is consistent with SESN2 expression. However, the decrease in SESN2 with the disease may be compromising the protective effect against diet, making dystrophic muscles further susceptible to IR-induced alterations in skeletal muscle. A disease-mediated reduction in SESN2 may have contributed at least partly to the increased inflammatory signaling and dysfunctional autophagy in dystrophic muscles (53, 84–87, 89).

Mice fed a HFHSD were hyperglycemic following fasting. This result is further supported by decreased 1,5-anhydroglucitol and glycine from the HFHSD-fed groups. 1,5-Anhydroglucitol is a dietary polyol and is an emerging biomarker of glycemic excursions (55, 90). The distinct patterns in the heatmap of differentially expressed metabolites from quadriceps muscles (Supplemental Fig. 3*A*) suggest metabolic dysregulation caused by HFHSD, dystrophin deficiency, and the combination of the HFHSD in dystrophin-deficient muscle. Intriguingly, glycine, an anti-inflammatory amino acid that is generally decreased with IR (91), was also decreased in mdx mice compared with C57 mice. Glycine supplementation has improved muscle function and decreased fibrosis in prednisolone-treated dystrophic mice (92). Altered BCAA metabolism in C57 HFHSD compared with C57 CD was consistent with disrupted BCAA metabolism reported with IR (93). In total, DI-IR in C57 mice caused metabolic dysregulation of pathways like BCAA and glutathione metabolism. Since many of these common features were differentially expressed with dystrophin deficiency (in C57 CD vs. mdx CD) and diet (C57 CD vs. C57 HFHSD), similar disruptions in metabolism may be caused by DI-IR or dystrophin deficiency. Of note, 15 metabolites were differentially regulated uniquely in the mdx HFHSD group. These results are suggestive of metabolic dysregulations that are unique and common with disease and diet. Importantly, the altered metabolite profile in mdx HFHSD suggests an impact of diet in the dystrophic environment and raises the possibility of functional differences between dystrophic muscle and dystrophic/DI-IR muscle.

There are several limitations that should be considered when interpreting data presented herein. First, we determined the average food consumed for each animal using the total food consumed for each cage divided by the total number of animals. Although this allows some animals to consume more food than others, given the long-term nature of this study, the large effect sizes caused by diet, and the relatively small variation, the impact of potential differential feed intake on major outcomes seems limited. Furthermore, this group-housing approach avoids the problem of stress induced by single-housing social animals like mice. Second, although this investigation if focused on the effect of HFHSD feeding in dystrophic muscles, the genetic backgrounds of the control mice and the mdx mice were different. The use of mdx and C57 mice with the same background may enhance the magnitude and resolution of physiological and metabolic alterations that are observed, though it is possible that differences between genotypes are due to lineage and not dystrophin deficiency. In addition, the effect of glucocorticoids, a common therapeutic intervention for DMD, in combination with a HFHSD would increase the translatability of this work.

### Perspectives and Significance

In total, this study provides the first evidence that HFHSD increased adipose deposition, glucose intolerance, and IR in mdx mice. DI-IR did not augment histological indices of muscle injury or cellular dysfunction; however, the HFHSD caused a unique metabolic shift in healthy and dystrophin-deficient skeletal muscle indicating that some underlying mechanisms have been impacted. Furthermore, in dystrophic skeletal muscle, how DI-IR may impair mitochondrial function and muscle function, particularly specific tension and fatigue resistance, remains unknown. Finally, given the high frequency of IR in boys/men with DMD, it may be important, and perhaps more predictive of clinical outcomes, to consider therapeutics and interventions within this physiological and metabolic context.

## DATA AVAILABILITY

The raw data used for lipidomics and metabolomics can be made available upon request.

## SUPPLEMENTAL DATA

10.25380/iastate.21280455 Supplemental Figs. S1–S3 and Table S1: https://doi.org/10.25380/iastate.21280455.

## GRANTS

This work was supported in part by a Faculty Seed Grant from the College of Human Sciences at ISU (to J.T.S. and R.J.V.) and M.Q. is supported by National Institutes of Health Grants DK121875, HL158531, DK130908, and AG078174 by and start-up funds, Trustee Award, Heart Institute Translational Funds, and CuSTOM pilot grant from Cincinnati Children’s Hospital Medical Center.

## DISCLOSURES

No conflicts of interest, financial or otherwise, are declared by the authors.

## AUTHOR CONTRIBUTIONS

S.K., C.H.R., R.J.V., and J.T.S. conceived and designed research; S.K., K.G.E., C.H.R., H.E., M.W., M.Q., R.J.V., and J.T.S. performed experiments; S.K., M.W., M.Q., R.J.V., and J.T.S. analyzed data; S.K., R.J.V., and J.T.S. interpreted results of experiments; S.K. prepared figures; S.K. drafted manuscript; S.K., K.G.E., C.H.R., H.E., M.W., M.Q., R.J.V., and J.T.S. edited and revised manuscript; S.K., K.G.E., C.H.R., M.W., M.Q., R.J.V., and J.T.S. approved final version of manuscript.

## References

[1] Bushby K, Finkel R, Birnkrant DJ, Case LE, Clemens PR, Cripe L, Kaul A, Kinnett K, McDonald C, Pandya S, Poysky J, Shapiro F, Tomezsko J, Constantin C; DMD Care Considerations Working Group. Diagnosis and management of Duchenne muscular dystrophy, part 1: diagnosis, and pharmacological and psychosocial management. Lancet Neurol 9: 77–93, 2010. doi:10.1016/S1474-4422(09)70271-6. 19945913

[2] Emery AE. Population frequencies of inherited neuromuscular diseases—a world survey. Neuromuscul Disord 1: 19–29, 1991. doi:10.1016/0960-8966(91)90039-u. 1822774

[3] De Palma C, Morisi F, Cheli S, Pambianco S, Cappello V, Vezzoli M, Rovere-Querini P, Moggio M, Ripolone M, Francolini M, Sandri M, Clementi E. Autophagy as a new therapeutic target in Duchenne muscular dystrophy. Cell Death Dis 3: e418, 2012. doi:10.1038/cddis.2012.159. 23152054 PMC3542595

[4] Hollinger K, Gardan-Salmon D, Santana C, Rice D, Snella E, Selsby JT. Rescue of dystrophic skeletal muscle by PGC-1α involves restored expression of dystrophin-associated protein complex components and satellite cell signaling. Am J Physiol Regul Integr Comp Physiol 305: R13–R23, 2013. doi:10.1152/ajpregu.00221.2012. 23594613

[5] Krishna S, Spaulding HR, Quindry TS, Hudson MB, Quindry JC, Selsby JT. Indices of defective autophagy in whole muscle and lysosome enriched fractions from aged D2-mdx mice. Front Physiol 12: 691245, 2021. doi:10.3389/fphys.2021.691245. 34305644 PMC8299564

[6] Ljubicic V, Jasmin BJ. AMP-activated protein kinase at the nexus of therapeutic skeletal muscle plasticity in Duchenne muscular dystrophy. Trends Mol Med 19: 614–624, 2013. doi:10.1016/j.molmed.2013.07.002. 23891277

[7] Moore TM, Lin AJ, Strumwasser AR, Cory K, Whitney K, Ho T, Ho T, Lee JL, Rucker DH, Nguyen CQ, Yackly A, Mahata SK, Wanagat J, Stiles L, Turcotte LP, Crosbie RH, Zhou Z. Mitochondrial dysfunction is an early consequence of partial or complete dystrophin loss in mdx mice. Front Physiol 11: 690, 2020. doi:10.3389/fphys.2020.00690. 32636760 PMC7317021

[8] Kim JH, Kwak HB, Thompson LV, Lawler JM. Contribution of oxidative stress to pathology in diaphragm and limb muscles with Duchenne muscular dystrophy. J Muscle Res Cell Motil 34: 1–13, 2013. doi:10.1007/s10974-012-9330-9. 23104273

[9] Petrillo S, Pelosi L, Piemonte F, Travaglini L, Forcina L, Catteruccia M, Petrini S, Verardo M, D'Amico A, Musarò A, Bertini E. Oxidative stress in Duchenne muscular dystrophy: focus on the NRF2 redox pathway. Hum Mol Genet 26: 2781–2790, 2017. doi:10.1093/hmg/ddx173. 28472288

[10] Pauly M, Angebault-Prouteau C, Dridi H, Notarnicola C, Scheuermann V, Lacampagne A, Matecki S, Fauconnier J. ER stress disturbs SR/ER-mitochondria Ca2+ transfer: implications in Duchenne muscular dystrophy. Biochim Biophys Acta Mol Basis Dis 1863: 2229–2239, 2017. doi:10.1016/j.bbadis.2017.06.009. 28625916

[11] Hollinger K, Selsby JT. The physiological response of protease inhibition in dystrophic muscle. Acta Physiol (Oxf) 208: 234–244, 2013. doi:10.1111/apha.12114. 23648220

[12] Radley-Crabb HG, Marini JC, Sosa HA, Castillo LI, Grounds MD, Fiorotto A. Dystropathology increases energy expenditure and protein turnover in the mdx mouse model of duchenne muscular dystrophy. PLoS One. 9: e89277, 2014. doi:10.1038/s41467-020-14895-9. 24586653 PMC3929705

[13] Lapidos KA, Kakkar R, McNally EM. The dystrophin glycoprotein complex: signaling strength and integrity for the sarcolemma. Circ Res 94: 1023–1031, 2004. doi:10.1161/01.RES.0000126574.61061.25. 15117830

[14] Rodriguez-Cruz M, Atilano-Miguel S, Barbosa-Cortés L, Bernabé-García M, Almeida-Becerril T, Cárdenas-Conejo A, Del Rocío Cruz-Guzmán O, Maldonado-Hernández J. Evidence of muscle loss delay and improvement of hyperinsulinemia and insulin resistance in Duchenne muscular dystrophy supplemented with omega-3 fatty acids: a randomized study. Clin Nutr 38: 2087–2097, 2019. doi:10.1016/j.clnu.2018.10.017. 30420291

[15] Rodríguez-Cruz M, Sanchez R, Escobar RE, Cruz-Guzmán Odel R, López-Alarcón M, Bernabe García M, Coral-Vázquez R, Matute G, Velázquez Wong AC. Evidence of insulin resistance and other metabolic alterations in boys with Duchenne or Becker muscular dystrophy. Int J Endocrinol 2015: 867273, 2015. doi:10.1155/2015/867273. 26089900 PMC4452344

[16] Davidson ZE, Ryan MM, Kornberg AJ, Sinclair K, Cairns A, Walker KZ, Truby H. Observations of body mass index in Duchenne muscular dystrophy: a longitudinal study. Eur J Clin Nutr 68: 892–897, 2014. doi:10.1038/ejcn.2014.93. 24824013

[17] Saure C, Caminiti C, Weglinski J, de Castro Perez F, Monges S. Energy expenditure, body composition, and prevalence of metabolic disorders in patients with Duchenne muscular dystrophy. Diabetes Metab Syndr 12: 81–85, 2018. doi:10.1016/j.dsx.2017.08.006. 28869151

[18] Weber DR, Hadjiyannakis S, McMillan HJ, Noritz G, Ward LM. Obesity and endocrine management of the patient with Duchenne muscular dystrophy. Pediatrics 142, *Suppl* 2: S43–S52, 2018. doi:10.1542/peds.2018-0333F. 30275248 PMC6460463

[19] Zanardi MC, Tagliabue A, Orcesi S, Berardinelli A, Uggetti C, Pichiecchio A. Body composition and energy expenditure in Duchenne muscular dystrophy. Eur J Clin Nutr 57: 273–278, 2003. doi:10.1038/sj.ejcn.1601524. 12571659

[20] Berberoğlu HT, Acar-Tek N. Nutritional problems in patients with Duchenne muscular dystrophy. Biomed J Sci Tech Res 34: 27105–27111, 2021. doi:10.26717/BJSTR.2021.34.005610.

[21] Matthews E, Brassington R, Kuntzer T, Jichi F, Manzur AY. Corticosteroids for the treatment of Duchenne muscular dystrophy. Cochrane Database Syst Rev 2016: CD003725, 2016. doi:10.1002/14651858.CD003725.pub4. 27149418 PMC8580515

[22] Quattrocelli M, Zelikovich AS, Jiang Z, Peek CB, Demonbreun AR, Kuntz NL, Barish GD, Haldar SM, Bass J, McNally EM. Pulsed glucocorticoids enhance dystrophic muscle performance through epigenetic-metabolic reprogramming. JCI Insight 4: e132402, 2019. doi:10.1172/jci.insight.132402. 31852847 PMC6975267

[23] Payne ET, Yasuda N, Bourgeois JM, Devries MC, Rodriguez MC, Yousuf J, Tarnopolsky MA. Nutritional therapy improves function and complements corticosteroid intervention in mdx mice. Muscle Nerve 33: 66–77, 2006. doi:10.1002/mus.20436. 16149047

[24] Quattrocelli M, Zelikovich AS, Salamone IM, Fischer JA, McNally EM. Mechanisms and clinical applications of glucocorticoid steroids in muscular dystrophy. J Neuromuscul Dis 8: 39–52, 2021. doi:10.3233/JND-200556. 33104035 PMC7902991

[25] Coulton GR, Curtin NA, Morgan JE, Partridge TA. The mdx mouse skeletal muscle myopathy. II. Contractile properties. Neuropathol Appl Neurobiol 14: 299–314, 1988. doi:10.1111/j.1365-2990.1988.tb00890.x. 3221977

[26] Dangain J, Vrbova G. Muscle development in mdx mutant mice. Muscle Nerve 7: 700–704, 1984. doi:10.1002/mus.880070903. 6543918

[27] Manning J, O'Malley D. What has the mdx mouse model of duchenne muscular dystrophy contributed to our understanding of this disease? J Muscle Res Cell Motil 36: 155–167, 2015. doi:10.1007/s10974-015-9406-4. 25669899

[28] Radley-Crabb HG, Marini JC, Sosa HA, Castillo LI, Grounds MD, Fiorotto ML. Dystropathology increases energy expenditure and protein turnover in the mdx mouse model of duchenne muscular dystrophy. PLoS One 2014. 9(2): e89277. 24586653 10.1371/journal.pone.0089277 PMC3929705

[29] Milad N, White Z, Tehrani AY, Sellers S, Rossi FMV, Bernatchez P. Increased plasma lipid levels exacerbate muscle pathology in the mdx mouse model of Duchenne muscular dystrophy. Skelet Muscle 7: 19, 2017. doi:10.1186/s13395-017-0135-9. 28899419 PMC5596936

[30] Virtue S, Vidal-Puig A. GTTs and ITTs in mice: simple tests, complex answers. Nat Metab 3: 883–886, 2021. doi:10.1038/s42255-021-00414-7. 34117483

[31] Sousa LGO, Marshall AG, Norman JE, Fuqua JD, Lira VA, Rutledge JC, Bodine SC. The effects of diet composition and chronic obesity on muscle growth and function. J Appl Physiol (1985) 130: 124–138, 2021. doi:10.1152/japplphysiol.00156.2020. 33211595 PMC7944928

[32] Festuccia WT, Blanchard PG, Belchior T, Chimin P, Paschoal VA, Magdalon J, Hirabara SM, Simões D, St-Pierre P, Carpinelli A, Marette A, Deshaies Y. PPARγ activation attenuates glucose intolerance induced by mTOR inhibition with rapamycin in rats. Am J Physiol Endocrinol Physiol 306: E1046–E1054, 2014. doi:10.1152/ajpendo.00683.2013. 24619883

[33] Quattrocelli M, Wintzinger M, Miz K, Panta M, Prabakaran AD, Barish GD, Chandel NS, McNally EM. Intermittent prednisone treatment in mice promotes exercise tolerance in obesity through adiponectin. J Exp Med 219: e20211906, 2022. doi:10.1084/jem.20211906. 35363257 PMC8980841

[34] Fahy E, Subramaniam S, Murphy RC, Nishijima M, Raetz CR, Shimizu T, Spener F, van Meer G, Wakelam MJ, Dennis EA. Update of the LIPID MAPS comprehensive classification system for lipids. J Lipid Res 50, Suppl: S9–S14, 2009. doi:10.1194/jlr.R800095-JLR200. 19098281 PMC2674711

[35] Spaulding HR, Ludwig AK, Hollinger K, Hudson MB, Selsby JT. PGC-1α overexpression increases transcription factor EB nuclear localization and lysosome abundance in dystrophin-deficient skeletal muscle. Physiol Rep 8: e14383, 2020. doi:10.14814/phy2.14383. 32109352 PMC7048376

[36] Fiehn O, Kopka J, Trethewey RN, Willmitzer L. Identification of uncommon plant metabolites based on calculation of elemental compositions using gas chromatography and quadrupole mass spectrometry. Anal Chem 72: 3573–3580, 2000. doi:10.1021/ac991142i. 10952545

[37] Koek MM, Muilwijk B, van der Werf MJ, Hankemeier T. Microbial metabolomics with gas chromatography/mass spectrometry. Anal Chem 78: 1272–1281, 2006 [Erratum in *Anal Chem* 78: 3839, 2006]. doi:10.1021/ac051683+. 16478122

[38] Vieira VJ, Valentine RJ, Wilund KR, Antao N, Baynard T, Woods JA. Effects of exercise and low-fat diet on adipose tissue inflammation and metabolic complications in obese mice. Am J Physiol Endocrinol Physiol 296: E1164–E1171, 2009. doi:10.1152/ajpendo.00054.2009. 19276393 PMC2681303

[39] Jain R, Wade G, Ong I, Chaurasia B, Simcox J. Determination of tissue contributions to the circulating lipid pool in cold exposure via systematic assessment of lipid profiles. J Lipid Res 63: 100197, 2022. doi:10.1016/j.jlr.2022.100197. 35300982 PMC9234243

[40] Castellano-Escuder P, González-Domínguez R, Carmona-Pontaque F, Andrés-Lacueva C, Sánchez-Pla A. POMAShiny: a user-friendly web-based workflow for metabolomics and proteomics data analysis. PLoS Comput Biol 17: e1009148, 2021. doi:10.1371/journal.pcbi.1009148. 34197462 PMC8279420

[41] Wickham H, Averick M, Bryan J, Chang W, McGowan L, François R, Grolemund G, Hayes A, Henry L, Hester J, Kuhn M, Pedersen T, Miller E, Bache S, Müller K, Ooms J, Robinson D, Seidel D, Spinu V, Takahashi K, Vaughan D, Wilke C, Woo K, Yutani H. Welcome to the Tidyverse. J Open Stat Softw 4: 1686, 2019. doi:10.21105/joss.01686.

[42] Fox J, Weisberg S. An R Companion to Applied Regression (3rd ed.). Thousand Oaks, CA: Sage Publications, 2019.

[43] Searle SR, Speed FM, Milliken GA. Population marginal means in the linear model: an alternative to least squares means. Am Stat 34: 216–221, 1980. doi:10.2307/2684063.

[44] Villanueva RAM, Chen ZJ and Job J. ggplot2: elegant graphics for data analysis. In: Measurement: Interdisciplinary Research and Perspectives (2nd ed.). 2019, vol. 17, p. 160–167. doi:10.1080/15366367.2019.1565254.

[45] Pang Z, Chong J, Zhou G, de Lima Morais DA, Chang L, Barrette M, Gauthier C, Jacques P-É, Li S, Xia J. MetaboAnalyst 5.0: narrowing the gap between raw spectra and functional insights. Nucleic Acids Res 49: W388–W396, 2021. doi:10.1093/nar/gkab382. 34019663 PMC8265181

[46] Bardou P, Mariette J, Escudié F, Djemiel C, Klopp C. jvenn: an interactive Venn diagram viewer. BMC Bioinformatics 15: 293, 2014. doi:10.1186/1471-2105-15-293. 25176396 PMC4261873

[47] Kim JJ, Sears DD. TLR4 and insulin resistance. Gastroenterol Res Pract 2010: 212563, 2010. doi:10.1155/2010/212563. 20814545 PMC2931384

[48] Giordano C, Mojumdar K, Liang F, Lemaire C, Li T, Richardson J, Divangahi M, Qureshi S, Petrof BJ. Toll-like receptor 4 ablation in mdx mice reveals innate immunity as a therapeutic target in Duchenne muscular dystrophy. Hum Mol Genet 24: 2147–2162, 2015. doi:10.1093/hmg/ddu735. 25552658 PMC4380065

[49] Spaulding HR, Kelly EM, Quindry JC, Sheffield JB, Hudson MB, Selsby JT. Autophagic dysfunction and autophagosome escape in the mdx mus musculus model of Duchenne muscular dystrophy. Acta Physiol 222: 1–11, 2018. doi:10.1111/apha.12944. 28834378

[50] Li H, Liu S, Yuan H, Niu Y, Fu L. Sestrin 2 induces autophagy and attenuates insulin resistance by regulating AMPK signaling in C2C12 myotubes. Exp Cell Res 354: 18–24, 2017. doi:10.1016/j.yexcr.2017.03.023. 28300563

[51] Codogno P, Meijer AJ. Autophagy: a potential link between obesity and insulin resistance. Cell Metab 11: 449–451, 2010. doi:10.1016/j.cmet.2010.05.006. 20519116

[52] Zhou L, Zhang J, Fang Q, Liu M, Liu X, Jia W, Dong LQ, Liu F. Autophagy-mediated insulin receptor down-regulation contributes to endoplasmic reticulum stress-induced insulin resistance. Mol Pharmacol 76: 596–603, 2009. doi:10.1124/mol.109.057067. 19541767 PMC2730390

[53] Lee Jun H, Budanov Andrei V, Talukdar S, Park Eek J, Park Hae L, Park HW, Bandyopadhyay G, Li N, Aghajan M, Jang I, Wolfe Amber M, Perkins Guy A, Ellisman Mark H, Bier E, Scadeng M, Foretz M, Viollet B, Olefsky J, Karin M. Maintenance of metabolic homeostasis by Sestrin2 and Sestrin3. Cell Metab 16: 311–321, 2012. doi:10.1016/j.cmet.2012.08.004. 22958918 PMC3687365

[54] Rhee SG, Bae SH. The antioxidant function of sestrins is mediated by promotion of autophagic degradation of Keap1 and Nrf2 activation and by inhibition of mTORC1. Free Radic Biol Med 88: 205–211, 2015. doi:10.1016/j.freeradbiomed.2015.06.007. 26117317

[55] Dungan KM, Buse JB, Largay J, Kelly MM, Button EA, Kato S, Wittlin S. 1,5-Anhydroglucitol and postprandial hyperglycemia as measured by continuous glucose monitoring system in moderately controlled patients with diabetes. Diabetes Care 29: 1214–1219, 2006. doi:10.2337/dc06-1910. 16731998

[56] Rodríguez-Cruz M, Cruz-Guzmán OR, Escobar RE, López-Alarcón M. Leptin and metabolic syndrome in patients with Duchenne/Becker muscular dystrophy. Acta Neurol Scand 133: 253–260, 2016. doi:10.1111/ane.12450. 26133644

[57] AbdelMassih AF, Esmail R, Zekri H, Kharabish A, ElKhashab K, Menshawey R, Ismail HA, Afdal P, Farid E, Affifi O. Revisiting the pathogenic role of insulin resistance in Duchenne muscular dystrophy cardiomyopathy subphenotypes. Cardiovasc Endocrinol Metab 9: 165–170, 2020. doi:10.1097/XCE.0000000000000203. 33225232 PMC7673770

[58] Tan-Chen S, Guitton J, Bourron O, Le Stunff H, Hajduch E. Sphingolipid metabolism and signaling in skeletal muscle: from physiology to physiopathology. Front Endocrinol (Lausanne) 11: 491, 2020. doi:10.3389/fendo.2020.00491. 32849282 PMC7426366

[59] Tsonaka R, Seyer A, Aartsma-Rus A, Spitali P. Plasma lipidomic analysis shows a disease progression signature in mdx mice. Sci Rep 11: 12993, 2021. doi:10.1038/s41598-021-92406-6. 34155298 PMC8217252

[60] McNally BD, Ashley DF, Hänschke L, Daou HN, Watt NT, Murfitt SA, MacCannell ADV, Whitehead A, Bowen TS, Sanders FWB, Vacca M, Witte KK, Davies GR, Bauer R, Griffin JL, Roberts LD. Long-chain ceramides are cell non-autonomous signals linking lipotoxicity to endoplasmic reticulum stress in skeletal muscle. Nat Commun 13: 1748, 2022. doi:10.1038/s41467-022-29363-9. 35365625 PMC8975934

[61] Valentine WJ, Mostafa SA, Tokuoka SM, Hamano F, Inagaki NF, Nordin JZ, Motohashi N, Kita Y, Aoki Y, Shimizu T, Shindou H. Lipidomic analyses reveal specific alterations of phosphatidylcholine in dystrophic mdx muscle. Front Physiol 12: 698166, 2021. doi:10.3389/fphys.2021.698166. 35095541 PMC8791236

[62] Stedman HH, Sweeney HL, Shrager JB, Maguire HC, Panettieri RA, Petrof B, Narusawa M, Leferovich JM, Sladky JT, Kelly AM. The mdx mouse diaphragm reproduces the degenerative changes of Duchenne muscular dystrophy. Nature 352: 536–539, 1991. doi:10.1038/352536a0. 1865908

[63] Frisard MI, McMillan RP, Marchand J, Wahlberg KA, Wu Y, Voelker KA, Heilbronn L, Haynie K, Muoio B, Li L, Hulver MW. Toll-like receptor 4 modulates skeletal muscle substrate metabolism. Am J Physiol Endocrinol Physiol 298: E988–E998, 2010. doi:10.1152/ajpendo.00307.2009. 20179247 PMC2867377

[64] Barros Maranhão J, de Oliveira Moreira D, Maurício AF, de Carvalho SC, Ferretti R, Pereira JA, Santo Neto H, Marques MJ. Changes in calsequestrin, TNF-α, TGF-β and MyoD levels during the progression of skeletal muscle dystrophy in mdx mice: a comparative analysis of the quadriceps, diaphragm and intrinsic laryngeal muscles. Int J Exp Pathol 96: 285–293, 2015. doi:10.1111/iep.12142. 26515458 PMC4693553

[65] Hotamisligil GS. Inflammatory pathways and insulin action. Int J Obes Relat Metab Disord 27, *Suppl* 3: S53–S55, 2003. doi:10.1038/sj.ijo.0802502. 14704746

[66] Rosenberg AS, Puig M, Nagaraju K, Hoffman EP, Villalta SA, Rao VA, Wakefield LM, Woodcock J. Immune-mediated pathology in Duchenne muscular dystrophy. Sci Transl Med 7: 299rv4, 2015. doi:10.1126/scitranslmed.aaa7322. 26246170 PMC5951380

[67] Hollinger K, Shanely RA, Quindry JC, Selsby JT. Long-term quercetin dietary enrichment decreases muscle injury in mdx mice. Clin Nutr 34: 515–522, 2015. doi:10.1016/j.clnu.2014.06.008. 24998094

[68] Evans NP, Misyak SA, Robertson JL, Bassaganya-Riera J, Grange RW. Dysregulated intracellular signaling and inflammatory gene expression during initial disease onset in Duchenne muscular dystrophy. Am J Phys Med Rehabil 88: 502–522, 2009. doi:10.1097/PHM.0b013e3181a5a24f. 19454857

[69] Acharyya S, Villalta SA, Bakkar N, Bupha-Intr T, Janssen PM, Carathers M, Li ZW, Beg AA, Ghosh S, Sahenk Z, Weinstein M, Gardner KL, Rafael-Fortney JA, Karin M, Tidball JG, Baldwin AS, Guttridge DC. Interplay of IKK/NF-kappaB signaling in macrophages and myofibers promotes muscle degeneration in Duchenne muscular dystrophy. J Clin Invest 117: 889–901, 2007. doi:10.1172/JCI30556. 17380205 PMC1821069

[70] Gordon BS, Kelleher AR, Kimball SR. Regulation of muscle protein synthesis and the effects of catabolic states. Int J Biochem Cell Biol 45: 2147–2157, 2013. doi:10.1016/j.biocel.2013.05.039. 23769967 PMC3759561

[71] Kemp GJ, Taylor DJ, Dunn JF, Frostick SP, Radda GK. Cellular energetics of dystrophic muscle. J Neurol Sci 116: 201–206, 1993. doi:10.1016/0022-510x(93)90326-t. 8393092

[72] Kuznetsov AV, Winkler K, Wiedemann F, von Bossanyi P, Dietzmann K, Kunz WS. Impaired mitochondrial oxidative phosphorylation in skeletal muscle of the dystrophin-deficient mdx mouse. Mol Cell Biochem 183: 87–96, 1998. doi:10.1023/a:1006868130002. 9655182

[73] Montgomery MK, Turner N. Mitochondrial dysfunction and insulin resistance: an update. Endocr Connect 4: R1–R15, 2015. doi:10.1530/EC-14-0092. 25385852 PMC4261703

[74] Sebori R, Kuno A, Hosoda R, Hayashi T, Horio Y. Resveratrol decreases oxidative stress by restoring mitophagy and improves the pathophysiology of dystrophin-deficient mdx mice. Oxid Med Cell Longev 2018: 9179270, 2018. doi:10.1155/2018/9179270. 30510631 PMC6231358

[75] Selsby JT, Morine KJ, Pendrak K, Barton ER, Sweeney HL. Rescue of dystrophic skeletal muscle by PGC-1α involves a fast to slow fiber type shift in the mdx mouse. PLoS One 7: e30063, 2012. doi:10.1371/journal.pone.0030063. 22253880 PMC3256197

[76] Qi Y, Ye Y, Wang R, Yu S, Zhang Y, Lv J, Jin W, Xia S, Jiang W, Li Y, Zhang D. Mitochondrial dysfunction by TFAM depletion disrupts self-renewal and lineage differentiation of human PSCs by affecting cell proliferation and YAP response. Redox Biol 50: 102248, 2022. doi:10.1016/j.redox.2022.102248. 35091324 PMC8802056

[77] Donadelli M, Dando I, Fiorini C, Palmieri M. UCP2, a mitochondrial protein regulated at multiple levels. Cell Mol Life Sci 71: 1171–1190, 2014. doi:10.1007/s00018-013-1407-0. 23807210 PMC11114077

[78] Schrauwen P, Hesselink M. UCP2 and UCP3 in muscle controlling body metabolism. J Exp Biol 205: 2275–2285, 2002. doi:10.1242/jeb.205.15.2275. 12110661

[79] Pauly M, Daussin F, Burelle Y, Li T, Godin R, Fauconnier J, Koechlin-Ramonatxo C, Hugon G, Lacampagne A, Coisy-Quivy M, Liang F, Hussain S, Matecki S, Petrof BJ. AMPK activation stimulates autophagy and ameliorates muscular dystrophy in the mdx mouse diaphragm. Am J Pathol 181: 583–592, 2012. doi:10.1016/j.ajpath.2012.04.004. 22683340

[80] Liu Y, Palanivel R, Rai E, Park M, Gabor TV, Scheid MP, Xu A, Sweeney G. Adiponectin stimulates autophagy and reduces oxidative stress to enhance insulin sensitivity during high-fat diet feeding in mice. Diabetes 64: 36–48, 2015. doi:10.2337/db14-0267. 25071026

[81] Ehrlicher SE, Stierwalt HD, Newsom SA, Robinson MM. Skeletal muscle autophagy remains responsive to hyperinsulinemia and hyperglycemia at higher plasma insulin concentrations in insulin-resistant mice. Physiol Rep 6: e13810, 2018. doi:10.14814/phy2.13810. 30047243 PMC6060106

[82] Morales-Scholz G, Howlett KF, Murphy RM, Kowalski GM, Bruce CR, Shaw CS. Autophagy modulation in the liver and skeletal muscle of high-fat fed mice. Proc Aus Physiol Soc 47: 124, 2016. http://aups.org.au/Proceedings/47/124P.

[83] Morales-Scholz MG, Swinton C, Murphy RM, Kowalski GM, Bruce CR, Howlett KF, Shaw CS. Autophagy is not involved in lipid accumulation and the development of insulin resistance in skeletal muscle. Biochem Biophys Res Commun 534: 533–539, 2021. doi:10.1016/j.bbrc.2020.11.048. 33261883

[84] Ben-Sahra I, Dirat B, Laurent K, Puissant A, Auberger P, Budanov A, Tanti JF, Bost F. Sestrin2 integrates Akt and mTOR signaling to protect cells against energetic stress-induced death. Cell Death Differ 20: 611–619, 2013. doi:10.1038/cdd.2012.157. 23238567 PMC3595485

[85] Bae Soo H, Sung Su H, Oh Sue Y, Lim Jung M, Lee Se K, Park Young N, Lee Hye E, Kang D, Rhee Sue G. Sestrins activate Nrf2 by promoting p62-dependent autophagic degradation of Keap1 and prevent oxidative liver damage. Cell Metab 17: 73–84, 2013. doi:10.1016/j.cmet.2012.12.002. 23274085

[86] Kim MG, Yang JH, Kim KM, Jang CH, Jung JY, Cho IJ, Shin SM, Ki SH. Regulation of Toll-like receptor-mediated Sestrin2 induction by AP-1, Nrf2, and the ubiquitin-proteasome system in macrophages. Toxicol Sci 144: 425–435, 2015. doi:10.1093/toxsci/kfv012. 25637945

[87] Yang JH, Kim KM, Kim MG, Seo KH, Han JY, Ka SO, Park BH, Shin SM, Ku SK, Cho IJ, Hwan Ki S. Role of sestrin2 in the regulation of proinflammatory signaling in macrophages. Free Radic Biol Med 78: 156–167, 2015. doi:10.1016/j.freeradbiomed.2014.11.002. 25463278

[88] Shin BY, Jin SH, Cho IJ, Ki SH. Nrf2-ARE pathway regulates induction of Sestrin-2 expression. Free Radic Biol Med 53: 834–841, 2012. doi:10.1016/j.freeradbiomed.2012.06.026. 22749810

[89] Kim JS, Ro SH, Kim M, Park HW, Semple IA, Park H, Cho US, Wang W, Guan KL, Karin M, Lee JH. Sestrin2 inhibits mTORC1 through modulation of GATOR complexes. Sci Rep 5: 9502, 2015 [Erratum in *Sci Rep* 5: 14029, 2015]. doi:10.1038/srep09502. 25819761 PMC4377584

[90] Kato A, Kunimatsu T, Yamashita Y, Adachi I, Takeshita K, Ishikawa F. Protective effects of dietary 1,5-anhydro-d-glucitol as a blood glucose regulator in diabetes and metabolic syndrome. J Agric Food Chem 61: 611–617, 2013. doi:10.1021/jf304683s. 23270454

[91] Yazdani A, Yazdani A, Saniei A, Boerwinkle E. A causal network analysis in an observational study identifies metabolomics pathways influencing plasma triglyceride levels. Metabolomics 12: 104, 2016. doi:10.1007/s11306-016-1045-2. 27330524 PMC4869741

[92] Ham DJ, Gardner A, Kennedy TL, Trieu J, Naim T, Chee A, Alves FM, Caldow MK, Lynch GS, Koopman R. Glycine administration attenuates progression of dystrophic pathology in prednisolone-treated dystrophin/utrophin null mice. Sci Rep 9: 12982, 2019. doi:10.1038/s41598-019-49140-x. 31506484 PMC6736947

[93] Newgard CB. Interplay between lipids and branched-chain amino acids in development of insulin resistance. Cell Metab 15: 606–614, 2012. doi:10.1016/j.cmet.2012.01.024. 22560213 PMC3695706

